# Grafting polymer brushes from nylon surfaces *via* hydrogen atom transfer

**DOI:** 10.1039/d6sc02508k

**Published:** 2026-05-29

**Authors:** Tyler E. Ball, Anna E. Ringuette, Julianna R. Koehl, Gozde Aktas Eken, Senthilkumar Duraivel, Christopher K. Ober, Yadong Wang, Geoffrey W. Coates, Brett P. Fors

**Affiliations:** a Department of Chemistry and Chemical Biology, Baker Laboratory, Cornell University Ithaca New York 14853-1301 USA brettfors@cornell.edu; b Department of Materials Science and Engineering, Cornell University Ithaca New York 14853-1801 USA; c Meinig School of Biomedical Engineering, College of Engineering, Cornell University Ithaca New York 14853-1801 USA

## Abstract

The direct functionalization of nylon surfaces with well-defined polymer brushes would enable access to functional materials for advanced biomedical and industrial applications. To this end, we developed a surface-initiated hydrogen atom transfer reversible addition–fragmentation chain-transfer (SI HAT-RAFT) polymerization to directly graft from nylon surfaces under mild conditions. Hydrogen abstraction by a triplet-excited thioxanthone catalyst initiates polymer chains, which are capped by a bistrithiocarbonate moiety. Our method is amenable to (meth)acrylic and acrylamide monomers and various commercially relevant nylon substrates, and we demonstrate spatial control over the polymerization by patterning nylon surfaces with polymer brushes. Finally, we explored the ability of our method to modify surface properties by measuring water contact angles with select polymer grafts and demonstrate that hydrophilic polymer brush modifications inhibit bovine serum albumin adsorption.

## Abstract 2

Surface-initiated HAT-RAFT enables direct functionalization of nylon surfaces with a diverse polymer scope to yield functional materials for advanced biomedical and industrial applications.

## Graphical abstract

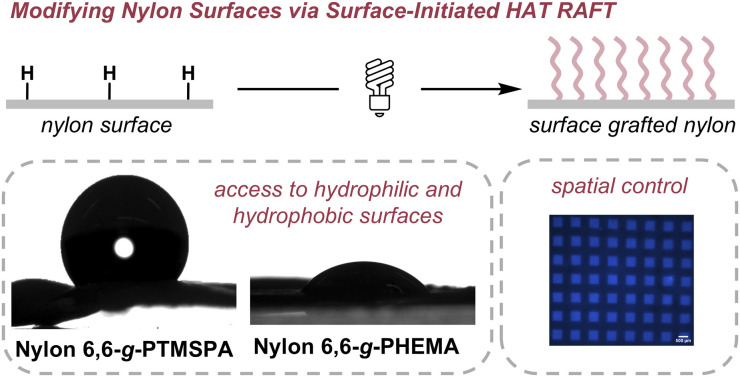

## Introduction

Aliphatic polyamides (*i.e.* nylons) are ubiquitous polymers with tuneable crystallinities,^1–3^ attractive material properties,^4^ and biocompatibility resulting from strong hydrogen bonding in the polyamide crystal structure.^5^ However, the applications of nylons as advanced materials are hindered by their surface wettability, biological inertness, and susceptibility to fouling.^6–8^ Functionalization of nylon surfaces is a key strategy to broaden the utility and robustness of these materials.

Various methods have been employed to chemically modify nylon surfaces, including plasma treatment,^6,9^ UV-irradiation,^10^*N*-alkylation,^11,12^ and polymer-graft modifications. Current methods for grafting from the surfaces of nylons and other polymeric surfaces are limited to uncontrolled polymerization strategies under harsh conditions^13–15^ or require lengthy synthetic routes to attach initiators/chain-transfer agents to polymer surfaces to achieve well-defined brushes.^8,16–20^ While diaryl ketones have been extensively studied as photosensitizers for surface functionalization, these studies have largely been limited to the initiation of free-radical polymerization *via* hydrogen atom transfer (HAT),^21^ require prior impregnation of the polymer surface with a photosensitizer,^22^ or graft a polymer network to the surface *via* C,H-insertion.^23–26^ A direct method of initiating controlled radical polymerization from nylon surfaces would be ideal.

**Scheme 1:**
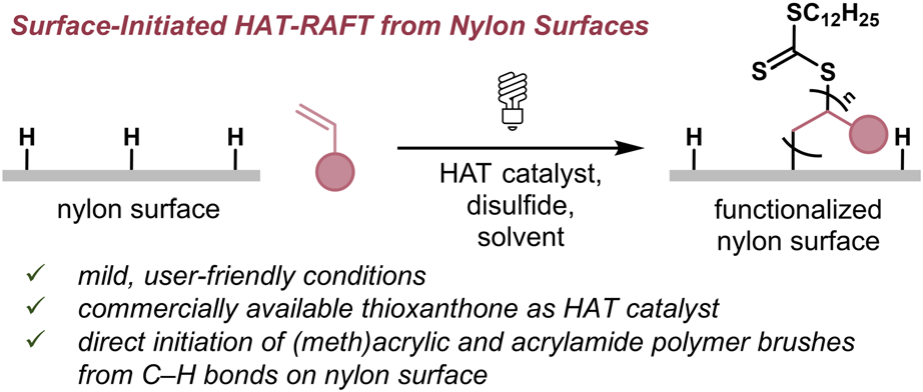
Surface-initiated HAT-RAFT *via* a thioxanthone derivative enables the direct functionalization of nylon surfaces.

In 2024, our group reported surface-initiated hydrogen atom transfer reversible addition–fragmentation chain-transfer (SI HAT-RAFT) polymerization for grafting from polyethylene surfaces.^27^ We found that under visible light irradiation in the presence of a benzophenone-derived HAT photocatalyst and a bis(trithiocarbonate) disulfide, thick and dense polymer brushes could be directly grafted from polyethylene surfaces. We posited that nylons would be amenable to SI HAT-RAFT due to the presence of hydridic hydrogen atoms on the polymer backbone which could be efficiently abstracted by the HAT catalyst to initiate polymerization. We envisaged that the wide scope of monomers amenable to SI HAT-RAFT would enable access to nylon materials with diverse surface properties. Herein, we develop a SI HAT-RAFT method as a mild, one-step method for functionalizing nylon surfaces (Scheme 1).

## Results and discussion

**Fig. 1:**
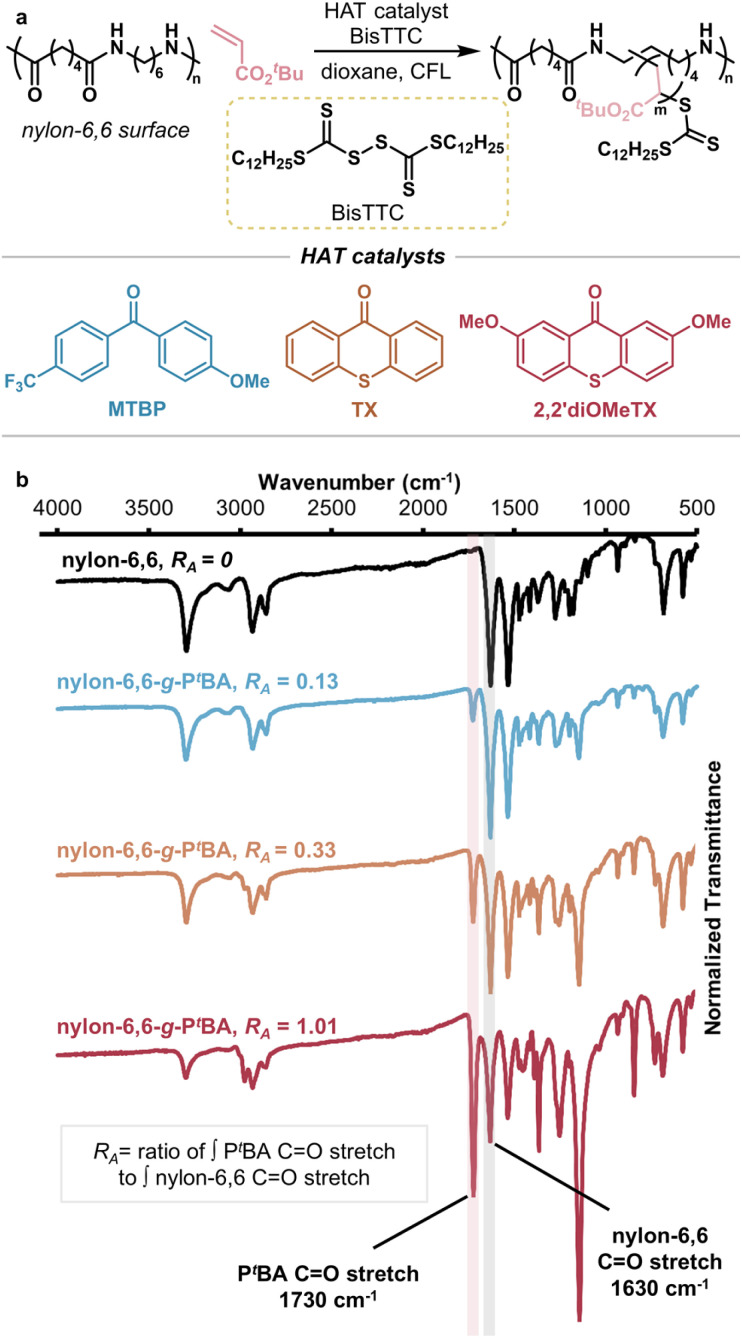
(a) General reaction scheme for the catalyst screen of SI HAT-RAFT from nylon-6,6: ^*t*^BA (200 equiv), HAT catalyst (1 equiv), and BisTTC (1 equiv) dissolved in dioxane (0.04 M in catalyst) and irradiated from 4 cm with a CFL in a nitrogen filled glovebox. (b) FT-IR spectra (normalised to the C=O stretch of nylon-6,6) of pristine nylon-6,6 (black) and nylon-6,6-*g*-P^*t*^BA generated after subjecting to SI HAT-RAFT conditions with MTBP (blue), TX (orange), and 2,2′diOMeTX (pink).

We began our investigation by screening a set of diaryl ketones as HAT catalysts for SI HAT-RAFT from nylon-6,6. A solvent-cast nylon-6,6 film was layered with a solution of HAT catalyst, bis-dodecyltrithiocarbonate disulfide (BisTTC), and ^*t*^butyl acrylate in 1,4-dioxane, covered with a glass slide, and irradiated with a compact fluorescent lamp (CFL) for 18 hours (Fig. 1a). After sonication of the irradiated films in dichloromethane to remove non-grafted polymer, FT-IR analysis revealed the appearance of a new carbonyl stretch at 1730 cm^−1^ corresponding to the C=O stretch of grafted poly(^*t*^butyl acrylate) (P^*t*^BA) (Fig. 1b). The ratio between the area of the C=O stretch of P^*t*^BA (1730 cm^−1^) and the C=O stretch of nylon-6,6 (1630 cm^−1^) (*R*_A_) was used as a qualitative proxy for grafting efficiency. Benzophenone-derived (4-methoxyphenyl)(4-(trifluoromethyl)phenyl)methanone (MTBP), which was the catalyst used for functionalization of polyethylene surfaces, yielded a modest amount of ^*t*^BA grafting (*R*_A_ = 0.13). We hypothesised that thioxanthone-derivatives would result in a greater degree of grafting than benzophenone-derivatives due to their relatively long triplet lifetimes.^28,29^ In support of this, switching to thioxanthone (TX) increased the amount of grafted ^*t*^BA significantly (*R*_A_ = 0.33). Commercially available 2,2′-dimethoxy-9*H*-thioxanthen-9-one (2,2′diOMeTX) resulted in the most intense FT-IR stretch after grafting with ^*t*^BA (*R*_A_ = 1.01); this is likely due to its increased absorbance in the visible region (*λ*_max_ = 415 nm) compared to TX,^30^ as the CFL has a strong emission at 435 nm (Fig. S1). Based on these high levels of grafting, 2,2′diOMeTX was used for further optimization.

To confirm that the observed polymer is covalently attached to the nylon-6,6 surface, nylon films were subject to a series of controls deviating from our standard SI HAT-RAFT conditions (Table 1, entry 1). When nylon-6,6 was dropcast with a solution of P^*t*^BA, the P^*t*^BA was washed away by sonication of the film in dichloromethane (Table 1, entry 2). Covalent attachment of the generated P^*t*^BA to nylon-6,6 after SI HAT-RAFT conditions was further verified by subjecting a film to standard reaction conditions and then subjecting to a Soxhlet extraction in THF, after which *R*_A_ remained 1.30 (Fig. S5).

**Table 1:** Optimization and control experiments

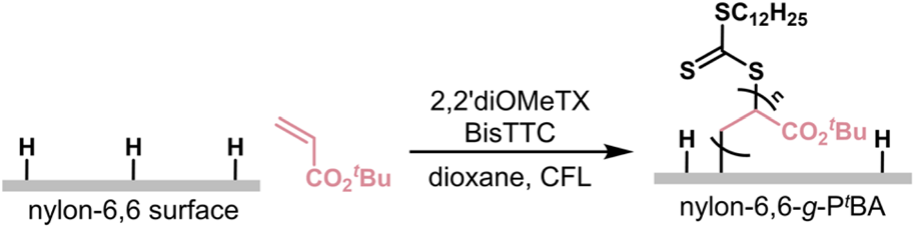		
Entry	Variation	*R*_A_
1^a^	Standard conditions	1.01
2^b^	Dropcast P^*t*^BA onto nylon-6,6	0.06
3^c^	PET-RAFT in the presence of nylon-6,6	0.02
4	No light	—
5	2,2′DiOMeTX removed	—
6	BisTTC removed	0.20
7	3 h irradiation	0.28
8^d^	Thermal RAFT chain extension from 7	0.53
9	Cyclohexane instead of 1,4-dioxane	0.23
10	DCE instead of 1,4-dioxane	0.16
11	Toluene instead of 1,4-dioxane	0.48
12	THF instead of 1,4-dioxane	0.65

a Standard conditions: ^*t*^BA (200 equiv), 2,2′diOMeTX (1 equiv), and BisTTC (1 equiv) dissolved in 1,4-dioxane (0.04 M in catalyst) and irradiated from 4 cm with a CFL in a nitrogen-filled glovebox for 18 h.

b Dropcast with 200 mg mL^−1^ P^*t*^BA (*M*_n_ = 30 kDa, *Đ* = 1.4) in DCM.

c Ir(ppy)_3_-catalysed PET-RAFT of ^*t*^BA in DMSO.

d Thermal RAFT chain extension from P^*t*^BA brushes using methyl acrylate (MA).

Next, we conducted experiments to probe the mechanism of SI HAT-RAFT. When Ir(ppy)_3_ catalysed photoinduced electron transfer RAFT (PET-RAFT) polymerization was conducted in the presence of a nylon-6,6 film (Table 1, entry 3) minimal P^*t*^BA grafting was observed, confirming that HAT is necessary for efficient polymer grafting and non-HAT radical generation is insufficient. Additionally, when subjected to standard conditions without light or without a photocatalyst, no grafting from nylon-6,6 was observed (Table 1, entries 4 and 5). Removal of the chain transfer agent precursor, BisTTC, does not completely inhibit grafting, but the intensity of the P^*t*^BA C=O stretch is diminished (Table 1, entry 6), suggesting that the bis(trithiocarbonate) is important for achieving efficient grafting. X-ray photoelectron spectroscopy (XPS) data taken after subjecting a sample to our standard conditions revealed that sulfur remained in the sample after washing with DCM (Fig. S21 and S22). Additionally, subjecting an SI HAT-RAFT grafted sample (Table 1, entry 7) to thermal RAFT chain extension conditions with AIBN and methyl acrylate (MA) (Table 1, entry 8) resulted in an increased *R*_A_ from 0.28 to 0.53, indicating that the disulfide species is capping polymer chains and can be used in chain extension reactions.

We theorise that a solvent with hydridic C–H bonds is necessary to provide a source of radicals in solution to enable efficient chain transfer with surface-bound chains.^27,31^ To investigate this, we screened solvents with a range of C–H electron densities and bond dissociation energies (Table 1, entries 9–12). Cyclohexane and dichloroethane (DCE), which lack activated C–H bonds (cyclohexane C–H BDE = 99 kcal mol^−1^)^29^ resulted in greatly diminished surface grafting compared to standard conditions with 1,4-dioxane. Toluene (C–H BDE = 88 kcal mol^−1^),^29^ with slightly activated benzylic hydrogens, showed increased surface grafting relative to unactivated solvents. In THF, which has C–H bonds of comparable electron-density to 1,4-dioxane and a lower BDE (92 kcal mol^−1^ compared to 96 kcal mol^−1^),^29^ surface grafting is only slightly diminished. Based on these results, we propose that a combination of the increased polarity of dioxane^22,28^ relative to unactivated solvents and the hydridic nature of the C–H bonds of dioxane aids in radical generation for the facilitation of chain transfer to promote chain growth from the nylon surface.

A proposed mechanistic scheme for SI HAT-RAFT from nylon-6,6 surfaces is provided in the SI (see Section S10 for details). We propose that visible light irradiation excites 2,2′diOMeTX to generate an electrophilic diradical, which abstracts a hydrogen from the nylon backbone to generate an initiating radical species. Small-molecule studies employing electrophilic radicals as HAT mediators from amides have reported chemoselectivity for the hydridic hydrogen atom α to the nitrogen;^32,33^ we posit that radical generation primarily occurs at this hydridic position, but abstraction from unactivated methylene units could also be occurring. Concurrent homolysis of bis(trithiocarbonate) disulfide by visible light yields two trithiocarbonyl radicals, one of which can combine with growing polymer chains. Importantly, without the ability to directly measure the dispersity of surface-attached polymer chains it is difficult to assess the degree of control attained by SI HAT-RAFT. While we have provided evidence that some population of surface-attached polymer chains are capped by a trithiocarbonate end-group, it is entirely possible that the surface polymerization is not well-controlled or that control is lost over time which may lead to branching.

We theorise that the kinetic selectivity of the electrophilic catalyst for electron-rich, hydridic hydrogen atoms precludes unwanted side reactivity, such as C–H abstraction from the electron-deficient polymer backbone.^34^ Our previous SI HAT-RAFT studies have shown that polymers with electron-poor, acidic C–H sites on the backbone are inaccessible.^27^ This is in accordance with our prior work grafting from small-molecules in solution to yield linear polymer chains^35^ as well as the lack of observable crosslinking in grafted nylon films (Fig. S24). We propose that the photocatalyst is regenerated by single-electron transfer and proton transfer with a trithiocarbonyl radical to generate trithiocarbonic acid, enabling continued radical generation.

**Fig. 2:**
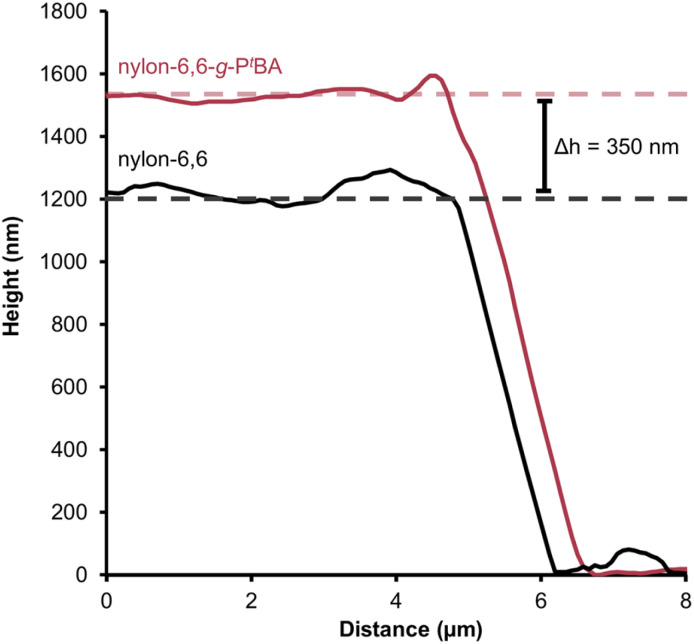
AFM measurement of nylon-6,6-*g*-P^*t*^BA. Brush thickness was assessed by comparing the depth of a scratch on a nylon-6,6 film before and after grafting under standard reaction conditions.

Having provided strong evidence that the P^*t*^BA brushes are covalently bound to the nylon surface, we sought to characterise P^*t*^BA brush thickness. Atomic force microscopy (AFM) was used to measure brush thicknesses *via* a change in step height before and after grafting. Silicon wafers spin-coated with nylon-6,6 in HFIP were subjected to standard reaction conditions to grow P^*t*^BA brushes. Under our optimised conditions with 2,2′diOMeTX, P^*t*^BA brushes were 350 nm thick (Fig. 2). This figure was verified by optical profilometry, in which brush thicknesses of 300 nm were measured (Fig. S25 and S26). Comparatively, ATRP polymerizations initiated from nylon 6,6 surfaces modified with alkyl bromide initiators report brush thicknesses of up to 80 nm ^36^ and RAFT polymerizations initiated from SiO_2_ surfaces generally report brush thicknesses below 100 nm.^19,31,37^ Due to the inability to directly evaluate polymer dispersity, it is possible that the thick graft layer observed in our system is a result of minimal control over polymerization. It is also possible that the thicker brushes obtained in our SI HAT-RAFT system are a result of the highly active thioxanthone catalyst and swelling of the nylon substrate by monomer and solvent leading to increased access to initiation sites. This is in agreeance with a polyethylene surface-grafting system wherein increased swelling time led to a greater percent grafting,^15^ as well as an ATRP system using surface-attached poly(vinyl chloride) as an initiator wherein it is proposed that thicker initial films resulted in increased graft thickness due to swelling of the substrate and increased access to initiation sites.^38^ Notably, pre-swelling a nylon-6,6 film with polymerization mixture for 14 hours prior to irradiation without excess solution present resulted in a similar degree of functionalization as standard reaction conditions for the same irradiation time (Fig. S29).

Based on the change in *R*_A_ observed upon switching the photocatalyst used for SI HAT-RAFT and the increase in *R*_A_ after subjecting to chain extension conditions, we sought to investigate whether brush thickness measurements would correlate with FT-IR intensities. Indeed, MTBP resulted in a P^*t*^BA brush thickness of 74 nm (*R*_A_ = 0.13) and TX in a brush thickness of 130 nm (*R*_A_ = 0.37) by optical profilometry (Fig. S30–S33), suggesting that *R*_A_ functions as a reasonable proxy for polymer brush thickness. Additionally, subjecting a spin-coated nylon-6,6 sample to SI HAT-RAFT conditions for 2 hours yielded a nylon-6,6-*g*-P^*t*^BA sample with a brush thickness of 33 nm by optical profilometry; a thermal RAFT chain extension resulted in an increased brush thickness to 88 nm (Fig. S34–36). This result aligns with the increase in *R*_A_ observed by FT-IR and indicates that some polymer chains are capped by a trithiocarbonate moiety.

**Fig. 3:**
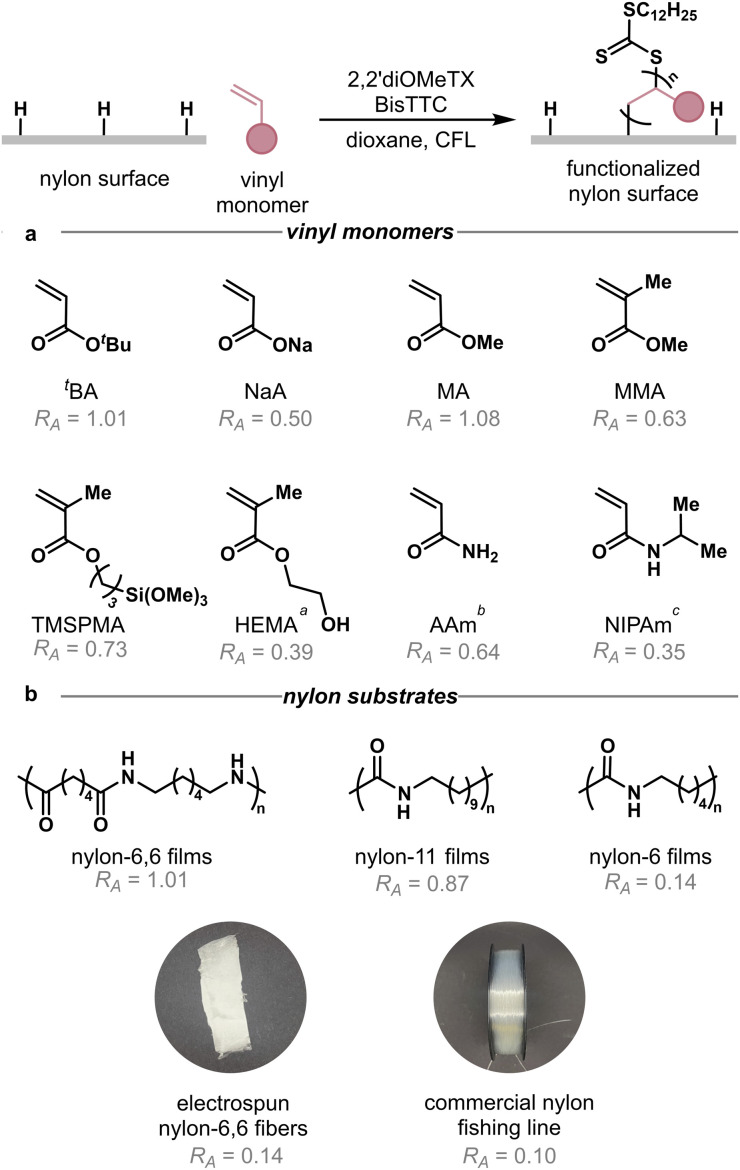
Scope of SI HAT-RAFT with (a) vinyl monomers and (b) various nylon substrates. ^*a*^Washed with DMF instead of DCM. ^*b*^DMSO/dioxane (50% v/v) used as solvent. *R*_A_ calculated as the ratio between the area of N–H stretch at 3183 cm^−1^ before and after grafting. ^*c*^*R*_A_ calculated as the ratio between the area of the C–H stretch at 2970 cm^−1^ before and after grafting.

To investigate the breadth of polymer brushes accessible *via* SI HAT-RAFT, nylon-6,6 was subjected to our standard conditions with a diverse set of vinyl monomers (Fig. 3a). (Meth)acrylic brushes are easily accessible under standard SI HAT-RAFT conditions; methyl acrylate (MA), methyl methacrylate (MMA), (trimethoxysilyl)propyl methacrylate (TMSPMA), and (hydroxyethyl)methacrylate (HEMA) brushes can be directly grafted from the surface of nylon-6,6. Additionally, P^*t*^BA can be hydrolysed under basic conditions to yield poly(sodium acrylate) (PNaA) brushes. Interestingly, polyacrylamide (PAAm) brushes have been shown to introduce flame-retardant characteristics to nylon-based textiles;^14,39^ as a result, we were curious if SI HAT-RAFT would be amenable to PAAm brush growth. After subjecting nylon-6,6 to modified SI HAT-RAFT conditions with AAm in a mixture of DMSO and dioxane, we observed the appearance of a characteristic PAAm N–H stretch at 3183 cm^−1^ by FT-IR (Fig. S43) indicating successful grafting. Due to their thermoresponsive nature,^13,40,41^ we were also interested in polymerizing poly(*N*-isopropylacrylamide) (PNIPAm) brushes. Standard reaction conditions with NIPAm yielded nylon-6,6-*g*-PNIPAm, based on the appearance of a characteristic N–H stretch at 2970 cm^−1^ and C–H bend at 1385 cm^−1^ (Fig. S45 and S46). Due to the high selectivity of electrophilic diaryl ketones for hydridic C–H bonds,^34^ monomers with electron-rich, hydridic C–H positions, such as HEMA and NIPAm, may result in a branched brush morphology or a crosslinked network. The polymer brushes reported here showcase the utility of SI HAT-RAFT for accessing a broad range of functional groups.

Next, we turned our attention to the scope of the nylon-surface (Fig. 3b). Interestingly, P^*t*^BA brushes can be efficiently initiated from nylon-11 surfaces, whereas nylon-6 resulted in a significantly depressed *R*_A_. We theorise that the decreased grafting observed from solvent-cast nylon-6 compared to nylon-11 and nylon-6,6 could be due to slight differences in crystallinity. Electrospun nylon-6,6 fibres, used in composites for filtration^42^ and for biomedical applications,^43,44^ were also accessible and SEM images revealed that SI HAT-RAFT reaction conditions did not impact the integrity of the fibres (Fig. S50). Interestingly, electrospinning nylon fibres has been reported to result in a predominately γ-crystal structure compared to the α-structure obtained from solvent-casting.^45,46^ Further studies relating substrate crystallinity and processing conditions to grafting efficiency are warranted. Subjecting commercially available nylon fishing line to SI HAT-RAFT conditions resulted in grafting (*R*_A_ = 0.10), but was less efficient, likely due to the presence of radical inhibitors in commercial samples.

**Fig. 4:**
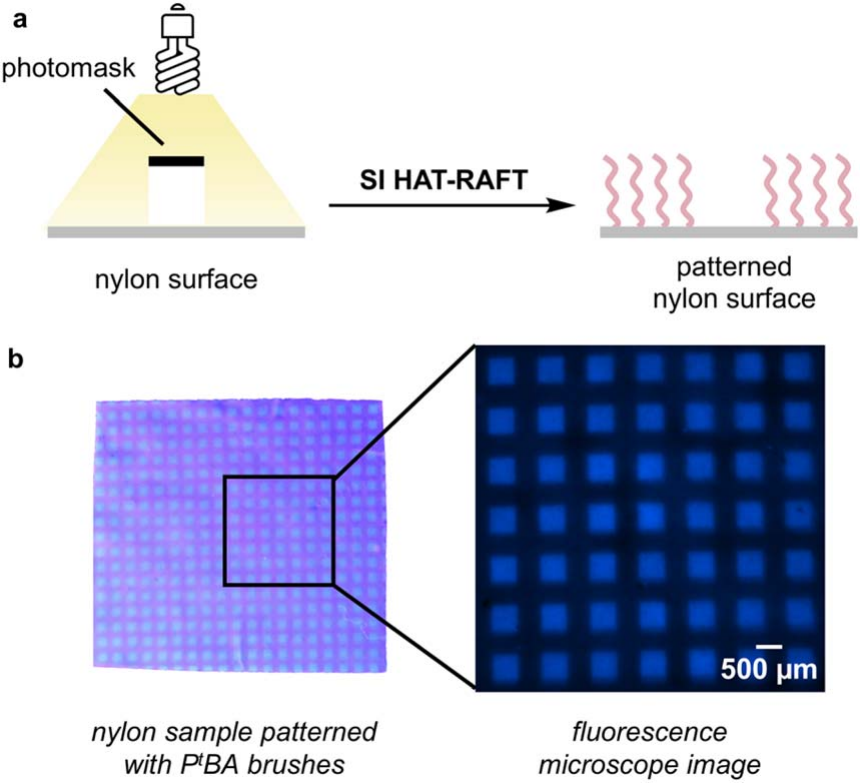
(a) Scheme depicting the spatial control attainable using SI HAT-RAFT when layered with a photomask. (b) Patterned nylon-11-*g*-P^*t*^BA surface imaged under 390 nm light and under a fluorescence microscope.

An advantage of using light to initiate controlled surface grafting is the ability to exert spatial control over the polymerization. To probe the potential of SI HAT-RAFT to pattern surfaces with polymer brushes, we subjected a nylon-11 film to SI HAT-RAFT conditions with ^*t*^BA and covered the sample with a photomask patterned with 500 by 500 µm transparent squares, as depicted in Fig. 4a. Upon washing the sample and irradiating with 390 nm light, we were able to visualise the patterned brushes macroscopically and under a fluorescence microscope (Fig. 4b). FT-IR measurements confirmed that brush growth only occurred where the sample was irradiated (Fig. S57) and we hypothesise that the observed fluorescence can be attributed to trace amounts of photocatalyst. Having verified the ability to exert spatial control over the polymerization, we envision that SI HAT-RAFT could be used to access complex surface architectures *via* patterning of polymer surface modifications.

**Fig. 5:**
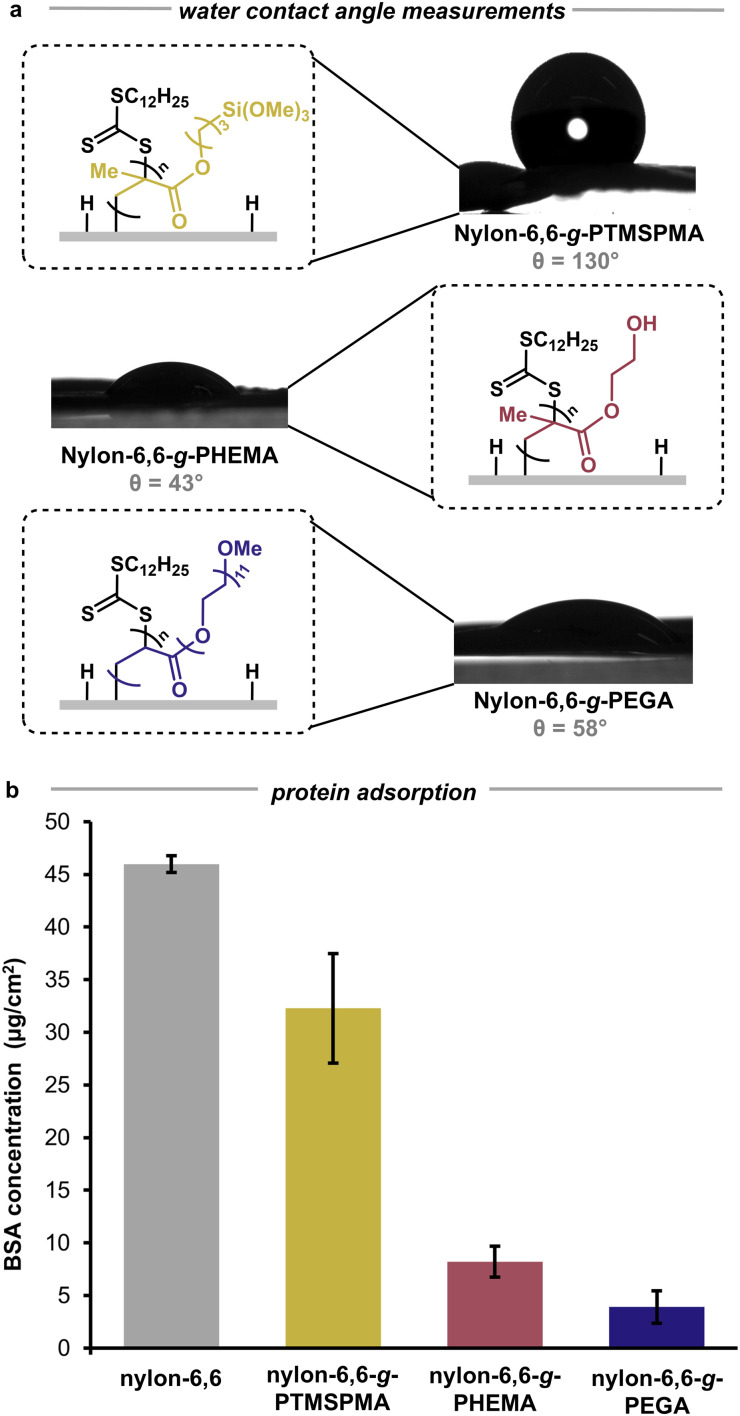
(a) Nylon-6,6-*g*-PTMSPMA, nylon-6,6-*g*-PHEMA, and nylon-6,6-*g*-PEGA water contact angle measurements. (b) BSA adsorption on a pristine nylon surface and nylon surfaces modified with hydrophobic and hydrophilic polymer brushes.

The hydrophilicity of nylon surfaces has been reported to influence the fouling and adhesive behaviour of the material.^47–49^ Water contact angles of select hydrophobic and hydrophilic polymer grafts were measured to showcase the ability of SI HAT-RAFT to alter the surface properties of nylons (Fig. 5a). PTMSPMA brushes increased the water contact angle (*θ*) of nylon-6,6 from 79° to 130°, whereas PHEMA and PEGA brushes resulted in hydrophilic surfaces (*θ* = 43° and 58°).

While nylons are commonly employed for biomedical applications, such as sutures and cell scaffolding, their utility is hindered by non-specific protein and cell adhesion.^17^ PHEMA and PEGA brushes have been reported to inhibit protein adhesion on polymer surfaces,^48^ and we theorised that hydrophobic PTMSPMA brushes would function as a positive control and would not inhibit protein adsorption due to the low surface energies of silicone surfaces.^49^ To test how polymer brushes introduced *via* SI HAT-RAFT alter protein adsorption, we submerged nylon 6,6-*g*-PTMSPMA, nylon 6,6-*g*-PHEMA, and nylon-6,6-*g*-PEGA in a solution of bovine serum albumin (BSA) (see Section S15 of SI for details). As depicted in Fig. 5b, the amount of adsorbed BSA slightly decreased from 46 ± 1 µg cm^−2^ on pristine nylon-6,6 to 32 ± 5 µg cm^−2^ on a PTMSPMA grafted sample. As expected, hydrophilic PHEMA and PEGA surface modifications greatly decreased the amount of protein that adhered to the substrate, with measured BSA concentrations of 8 ± 2 µg cm^−2^ and 4 ± 2 µg cm^−2^, respectively. This experiment demonstrates the potential for SI HAT-RAFT to modulate surface hydrophilicity and inhibit non-specific protein adsorption using BSA as a model protein.

## Conclusions

This study demonstrates the utility of SI HAT-RAFT for directly functionalizing nylon substrates with a diverse array of polymer brushes. Using a commercially available thioxanthone catalyst, we have obtained a P^*t*^BA brush thickness of ∼300 nm, which could result from either monomer swelling into the nylon substrate or a lack of control over the polymerization. We have shown that SI HAT-RAFT is amenable to both (meth)acrylic and acrylamide monomer classes and that we can graft from commercially relevant nylon substrates. We explored the application of SI HAT-RAFT to access nylon surfaces patterned with polymer brushes. Additionally, we showed that we could tune the hydrophilicity of the polymer surface by changing the monomer employed. Hydrophilic PHEMA and PEGA grafted samples resulted in greatly diminished BSA adsorption, whereas hydrophobic PTMSPMA only slightly decreased the amount of adsorbed protein. We envision that SI HAT-RAFT will enable access to functional nylon surfaces for biomedical and industrial applications.

## Author contributions

B. P. F. and G. W. C. supervised and provided guidance to the project. T. E. B. prepared all samples and analysed the data. A. E. R. carried out water contact angle measurements. J. R. K. conducted BSA adhesion tests. G. A. E. measured step heights *via* AFM measurements. S. D. obtained fluorescence microscope images of patterned nylon substrates. Y. W. and C. K. O. provided the necessary instrumentation and supervision for characterization of polymer brushes. T. E. B. prepared the manuscript and SI.

## Conflicts of interest

There are no conflicts to declare.

## Supplementary Material

SC-017-D6SC02508K-s001

## Data Availability

General reagent and analytical information, synthesis and polymerisation procedures, and surface characterisation data supporting this study have been included as part of the supplementary information (SI). Supplementary information is available. See DOI: https://doi.org/10.1039/d6sc02508k.
