# Supplementary material for: Grafting polymer brushes from nylon surfaces via hydrogen atom transfer
Source: Chem Sci. 2026 May 29;17(28):13945–51. doi: 10.1039/d6sc02508k (PMC13251061; doi:10.1039/d6sc02508k)
Supplement: SC-017-D6SC02508K-s001 [file SC-017-D6SC02508K-s001.pdf]

Supplementary Information (SI) for Chemical Science.
This journal is © The Royal Society of Chemistry 2026

Grafting Polymer Brushes from Nylon Surfaces via Hydrogen Atom Transfer

Tyler E. Ball, Anna E. Ringuette, Julianna Koehl, Gozde Aktas Eken, Senthilkumar Duraivel, Christopher K. Ober, Yadong Wang, Geoffrey W. Coates,* Brett P. Fors*

Cornell University, Ithaca, New York, 14853, United States

Supporting Information

Experimental Procedures

S1. General Reagent Information 3
S2. General Analytical Information 3
S3. General Photochemical Information 4
S4. Synthesis of Reagents 6
S5. Preparation of Nylon substrates 6
S6. General Procedure for Surfaced Initiated HAT-RAFT 7
S7. Optimization and Control Experiments 11
S8. Synthesis of brush polymers for XPS and AFM studies 20
S9. XPS studies 20
S10. Discussion of proposed SI HAT-RAFT mechanism using 2,2'diOMeTX as a hydrogen atom abstraction catalyst 23
S11. Brush Height Analysis 25
S12. Synthesis of Brush Polymers for Monomer Scope 32
S13. Water Contact Angle Measurements 45
S14. Patterning Experiments 47
S15. Protein Adsorption Experiments 48

S1. General Reagent Information

Tert-butyl acrylate (^{t}BuA) (99%, Sigma Aldrich), methyl acrylate (MA) (99%, Sigma Aldrich), methyl methacrylate (MMA) (99%, Sigma Aldrich), vendor), (hydroxyethyl)methacrylate (HEMA) (97%, Beantown Chemical), 3-(trimethoxysilyl)propyl methacrylate (TMSPA) (95%, AstaTech), dimethyl sulfoxide (99%, Sigma Aldrich), toluene (99%, Fisher Scientific), tetrahydrofuran (99%, Fisher Scientific), cychlohexane (99%, Fisher Scientific), dichloroethane (99.8%, Sigma Aldrich) and 1,4, dioxane (99.8%, ThermoScientific) were dried over CaH_2, distilled, and degassed via 3 freeze-pump-thaw cycles prior to storing in a nitrogen-filled glovebox. *N*-isopropyl acrylamide (98%, Tokyo Chemical Industry America) was dissolved in dichloromethane, filtered through a plug of basic alumina, and concentrated under reduced pressure prior to use. PEG-acrylate (M_n=480, Sigma) was filtered through a plug of basic alumina prior to use. Acrylamide (99%, Beantown Chemical), 2-Cyanopropan-2-yl dodecyl carbonotrithioate (97%, Ambeed), tris(1-phenypyridne)iridium(III) (99%, Ambeed), 1,1,1,3,3,3-Hexafluoro-2-propanol (HFIP) (99%, Apollo Scientific), thioxanthone (98%. Tokyo Chemical Industry), 2,7-dimethoxy-9H-thioxanthen-9-one (98%, Ambeed), and nylon-6,6, nylon-11, and nylon-6 pellets (Sigma Aldrich) were used as received. Nylon monofilament (Trilene fishing wire, Berkley Fishing) was cut in to 3-inch pieces before use as a substrate.

Polymerizations were carried out under irradiation with a compact fluorescent light (SATCO S7334, 40 W, 120 V, 60 Hz, 2700 K, 2600 lumens). Glass microscope slides (25 mm x 75 mm x 1 mm) were purchased from VWR. A fused-silica photomask purchased from the Cornell Nano Facility (CNF) was used for photopatterning experiments.

S2. General Analytical Information

Fourier-transform (FT-IR) spectra were taken on a Bruker Tensor II IR spectrometer equipped with a diamond Attenuated Total Reflectance (ATR) attachment. Generally, FT-IR spectra were normalized to the C=O stretch of nylon at ~1630 cm^{-1}. The ratio between peak areas (R_A) was calculated by integrating the C=O stretch of the polymer brush at 1730 cm^{-1} and dividing by the integration of the C=O stretch of nylon-6,6 at 1630cm^{-1} after spectra normalization.

$$R_A = \frac{\int carbonyl\ stretch\ of\ brush}{\int carbonyl\ stretch\ of\ nylon}$$

Equation 1. R_A calculations for (meth)acrylic polymer brushes.

In the case polyacrylamide and poly(N-isopropyl acrylamide), where the carbonyl stretch of the polymer brush overlaps with that of nylon, FT-IR spectra were normalized to the N–H stretch of nylon-6,6 at 3295 cm^{-1}. The regions where characteristic features grow in (3116 to 3375 cm^{-1} for polyacrylamide and 2970 to 3000 cm^{-1} (poly(N-isopropyl acrylamide)) were integrated before and after grafting and the difference between these integrations was used to calculate R_A.

$$\boldsymbol{R_A = \frac{\int_{3116}^{3375} after\ grafting - \int_{3116}^{3375} before\ grafting}{\int_{3116}^{3375} before\ grafting}}$$

Equation 2. R_A calculations for polyacrylamide brushes.

$$R_A = \frac{\int_{2970}^{3000} after\ grafting - \int_{2970}^{3000} before\ grafting}{\int_{2970}^{3000} before\ grafting}$$

Equation 3. R_A calculations for poly(N-isopropyl acrylamide) brushes.

Nuclear magnetic resonance (NMR) spectra were recorded on a Bruker 500 MHz instrument at room temperature using $CDCl_3$ as a solvent unless otherwise noted. Gel permeation chromatography (GPC) of the polymer sample used for dropcasting experiments was performed using a Tosoh EcoSEC HLC 8320 GPC system with two SuperHM-M columns in series at a flow rate of 0.35 mL/min. Tetrahydrofuran was used as the eluent and number-average molecular weights (M_n), weight-average molecular weights (M_w), and dispersities (Đ) were determined against polystyrene standards.

An Oxford Instruments Cypher ES atomic force microscope equipped with an environmental scanner was used for AFM measurements. Silicon tips with a resonance frequency of 300 kHz and a 26 N/m spring constant (Oxford Instruments) were used. To measure brush thickness, samples were scratched with a razor blade, and AFM height images were taken at the boundary between the scratched and non-scratched regions. Imaging was conducted in tapping mode, and the thickness of each sample was measured in five different regions. Brush thicknesses were also measured with a Keyence VK-X260 Laser-Scanning Profilometer. Samples of nylon-6,6 spin-coated onto a silicon wafer were scratched with a razor blade, the depth of the scratch was measured before and after brush growth, and the difference in scratch depth before and after functionalization was recorded as the brush thickness. Samples for XPS were analyzed using a Thermo Scientific Nexsa G2Spectrometer with operating pressure ca. 1x10-9 Torr. Monochromatic Al Kα x rays (1486.6 eV) with photoelectrons collected from a 400 µm diameter analysis spot. Photoelectrons were collected at a 90° emission angle with source to analyzer angle of 54.7°. A hemispherical analyzer determined electron kinetic energy, using a pass energy of 200 eV for wide/survey scans, and 50 eV for high resolution scans. A flood gun was used for charge neutralization of non-conductive samples. Static water contact angles were measured on a Rame-Hart 500 Goniometer using Milli-Q deionized water (15 µL). The average of at least 5 repetitive tests per sample was calculated. Fluorescence and brightfield images of the polymer brush patterns were collected using an inverted Nikon Eclipse Ti-2 microscope. All images were processed in Fiji.

S3. General Photochemical Information

Wavelengths of CFL Emission: The emission spectrum of the CFL employed in this study was recorded using an Ocean Optics USB200 spectrophotometer.

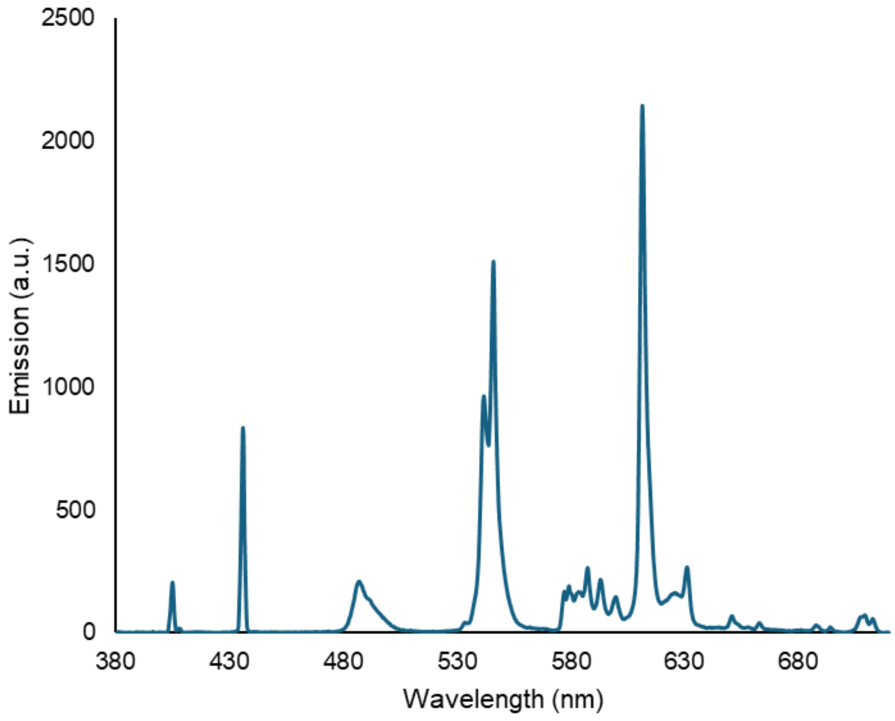

Figure S1. Emission spectrum of compact fluorescent light (Sylvania CF4OEL, 40 W, 120 V, 60 Hz, 2700 K, 2600 lumens).

Light intensity: The intensity of our CFL was measured using a ThorLabs USB Power Meter, Integrating Sphere Sensor, FC Fiber Adapter, Si, 350-1100 nm, 500 mW max. Using a 5 mm beam aperture 4 cm away from the CFL, the intensity of 610 nm and 540 nm wavelengths were measured to be 6.54 mW/cm2 and 8.64 mW/cm2, respectively.

Light temperature: The temperature of our CFL lamp was measured using a TOPDON TC005 mini thermal imaging camera. The lamp was turned on for 2 hours and covered in foil before the foil was quickly removed and the lamp temperature measured. The hottest temperature of the light was 119 °C, but the surrounding area remained close to room temperature.

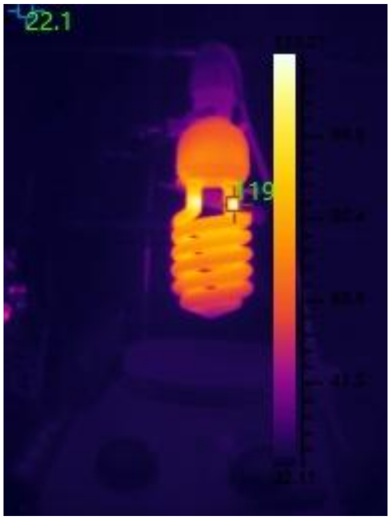

Figure S2. Thermal image of CFL and surrounding area immediately after removing aluminum foil.

Optical properties of glass slides: Borosilicate glass transmits from the infrared down to approximately 300 nm,[1] which is sufficiently transparent to allow the full emission spectrum of the CFL used in this study to reach the substrate.

S4. Synthesis of Reagents

Preparation of bistrithiocarbonate disulfide: Bis-dodecyltrithiocarbonate disulfide was prepared according to literature procedure.[2]

Preparation of MTBP (4-methoxyphenyl)(4-(trifluoromethyl)phenyl)methanone): MTBP was prepared according to literature procedure.[3]

S5. Preparation of Nylon substrates

Preparation of solvent-cast Nylon films

A 0.5 wt% solution of either nylon-6,6, nylon-11, or nylon-6 pellets in HFIP was stirred at room temperature until homogenous and layered into a glass petri dish. The glass petri dish was covered with a crystallizing glass dish and HFIP was allowed to evaporate until a smooth film remained. The films were then cut in to 2 cm by 2 cm pieces for use as substrates.

Preparation of spin-coated nylon-6,6 films

Using a glass Pasteur pipette, five drops of a 0.5 wt% solution of nylon-6,6 in HFIP were dropped on to a 1 cm by 1 cm piece of a thermal oxide silicon wafer affixed to a spin coater. The wafer was spun at 500 rpm for 60 seconds. Samples were annealed in a 110 °C oven overnight prior to use.

Preparation of electrospun nylon-6,6 fibers

Electrospun nylon-6,6 fibers were prepared according to a modified literature procedure.[4] A 10 wt% solution of nylon-6,6 in HFIP was stirred at room temperature until homogenous. The electrospinning apparatus consisted of a syringe pump (Harvard Apparatus PHD Ultra), a high-voltage power supply (Gamma High Voltage Research HV Power Supply ES30P-5W 30kV), and an aluminum plate collector. The nylon-6,6 solution was drawn in to a 6 mL syringe and attached to the syringe pump. A high voltage power supply was attached to the needle and a voltage of 20 kV was applied to the sample at a flow rate of 5 mL/h. Electrospun mats were collected on an aluminum plate 23 cm away from the syringe. Experiments were performed at 19.6 °C and 30% relative humidity. All samples were dried under ambient conditions and cut in to 2 cm by 2 cm pieces before use as substrates.

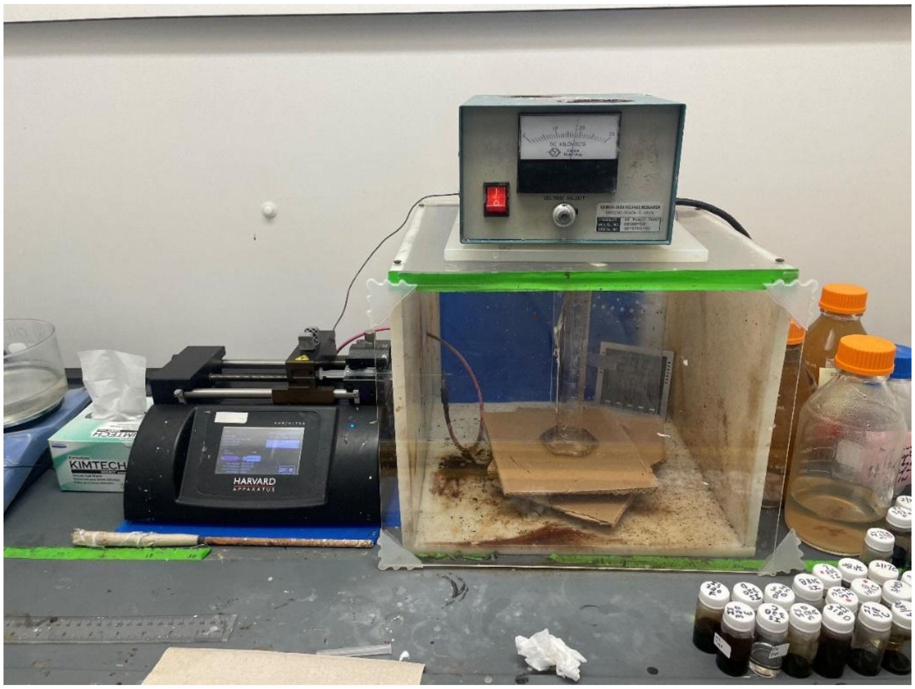

Figure S3. Image of electrospinning apparatus.

S6. General Procedure for Surfaced Initiated HAT-RAFT

Standard Reaction Conditions

In a nitrogen-filled glovebox, 2,2'diOMeTX (1 equiv, 0.01 mmol, 2.7 mg), BisTTC (1 equiv, 0.01 mmol, 5.5 mg), tert-butyl acrylate (200 equiv, 2 mmol, 0.29 mL), and solvent (0.25 mL) were combined in a one-dram vial. The solution was syringed onto a flat nylon (nylon-6,6, nylon-11, or nylon-6) substrate, which was placed on a glass microscope slide. The reaction mixture was covered with a second glass microscope slide and irradiated with a CFL

lamp placed 4 cm away without air cooling. The reaction was allowed to proceed for 18 hours before the light was turned off and the substrate removed from the glovebox. The substrate was sonicated in dichloromethane for 20 min prior to drying under ambient conditions.

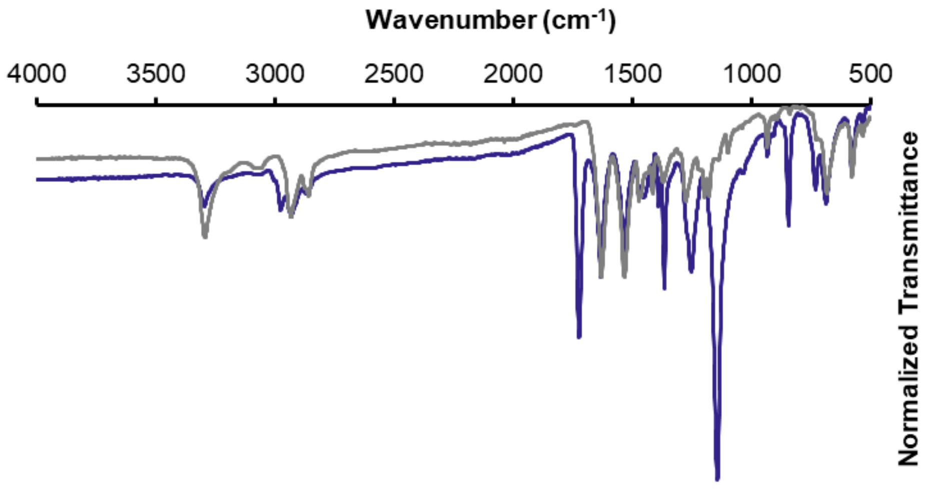

Figure S4. FT-IR spectra of nylon-6,6 before (gray) and after (purple) standard reaction conditions with P^{t}BA.

To further verify that any intercalating polymer chains were removed from the substrate, the sample was subjected to a Soxhlet extraction in THF at 70°C overnight. The resulting spectra revealed similar carbonyl stretch intensities at 1730 cm^{-1}, so sonication in dichloromethane was chosen as a washing method.

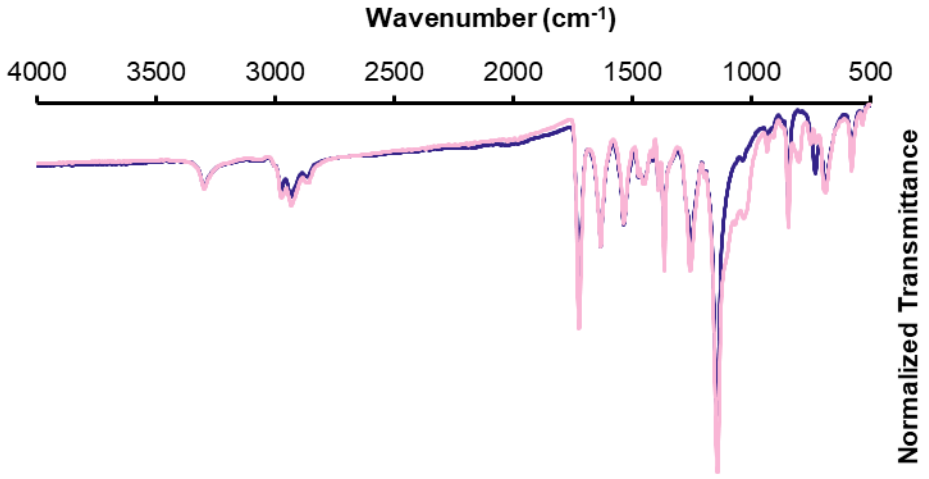

Figure S5. FT-IR spectra of nylon-6,6-*g*-P^{t}BA before (purple) and after (pink) a Soxhlet extraction in THF.

Standard conditions with MTBP as a photocatalyst

In a nitrogen-filled glovebox, MTBP (1 equiv, 0.01 mmol, 2.8 mg), BisTTC (1 equiv, 0.01 mmol, 5.5 mg), and *tert*-butyl acrylate (200 equiv, 2 mmol, 0.29 mL), and dioxane (0.25 mL) were combined in a one-dram vial. The solution was syringed onto a flat nylon-6,6 substrate, which was laid on a glass microscope slide. The reaction mixture was covered with a second glass microscope slide and irradiated with a CFL lamp placed 4 cm away without air cooling. The reaction was allowed to proceed for 18 hours before the light was turned off and the substrate removed from the glovebox. The substrate was sonicated in dichloromethane for 20 min prior to drying with air.

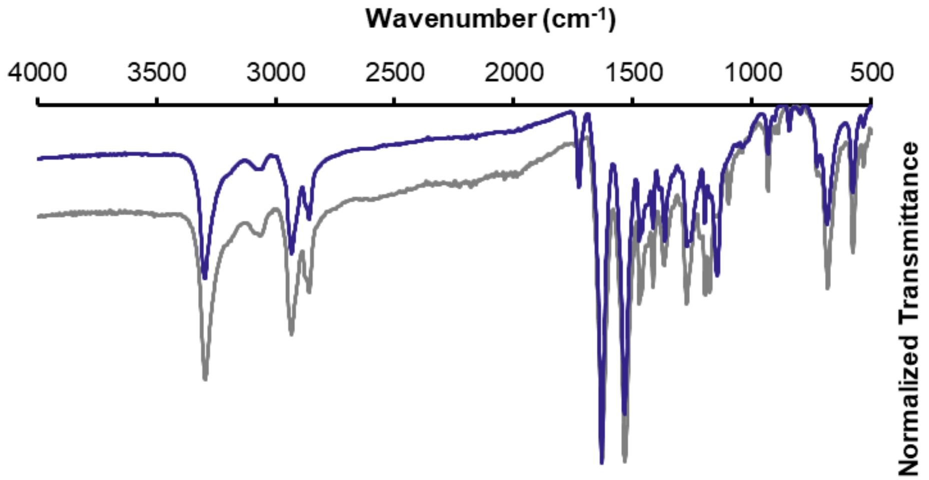

Figure S6. IR of nylon-6,6 before (gray) and after (purple) standard reaction conditions with MTBP as a photocatalyst.

Standard conditions with thioxanthone as a photocatalyst

In a nitrogen-filled glovebox, thioxanthone (1 equiv, 0.01 mmol, 2.1 mg), BisTTC (1 equiv, 0.01 mmol, 5.5 mg), and *tert*-butyl acrylate (200 equiv, 2 mmol, 0.29 mL), and dioxane (0.25 mL) were combined in a one-dram vial. The solution was syringed onto a flat nylon-6,6 substrate, which was laid on a glass microscope slide. The reaction mixture was covered with a second glass microscope slide and irradiated with a CFL lamp placed 4 cm away without air cooling. The reaction was allowed to proceed for 20 hours before the light was turned off and the substrate removed from the glovebox. The substrate was sonicated in dichloromethane for 20 min prior to drying with air.

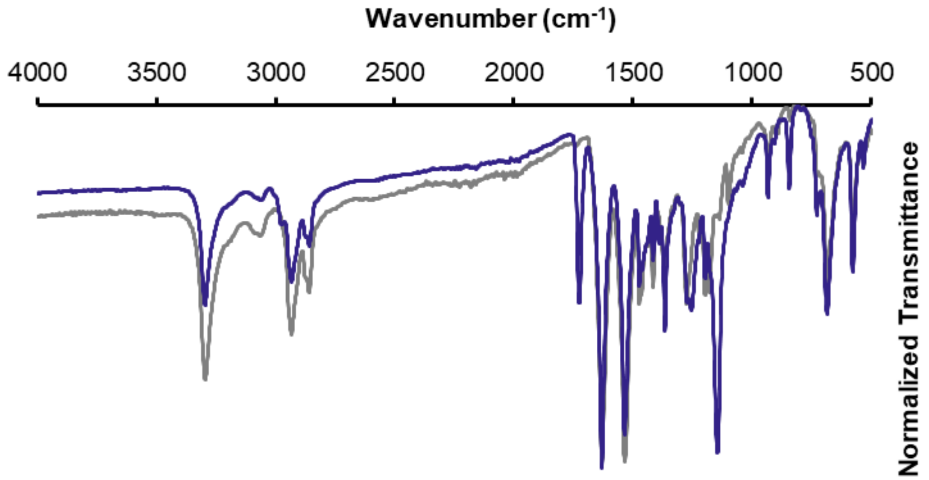

Figure S7. IR of nylon-6,6 before (gray) and after (purple) standard reaction conditions with thioxanthone as a photocatalyst.

S7. Optimization and Control Experiments

Drop-casting PtBA onto nylon-6,6

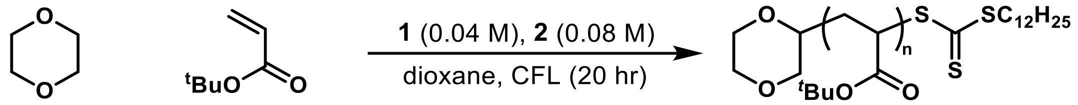

Poly(*tert*-butyl acrylate) (P*t*BA) samples were prepared according to published HAT-RAFT conditions.[3] MTBP (5.6 mg, 1 equiv, 0.01 mmol), BisTCC (11.1 mg, 2 equiv, 0.02 mmol), *tert*-butyl acrylate (0.59 mL, 200 equiv, 4 mmol), and dioxane (0.5 mL) were combined in a one-dram vial. The solution was freeze-pump-thawed a total of three times and placed 1 cm away from a CFL lamp with cooling. After 20 hours of irradiation, the reaction was opened to air and aliquots were taken for NMR and GPC analysis. Volatiles were removed in vacuo and the resulting polymer was purified by precipitating three times in cold methanol. ^{1}H NMR: 99% conversion, M_n^{theo} = 26.1 kg/mol. GPC = M_n^{RI}=29.5 kg/mol, Đ=1.4.

A 200 mg/mL solution of the prepared P*t*BA sample in dichloromethane was prepared and layered on to a solvent-cast nylon-6,6 film. After evaporation of the solvent overnight, the substrate was sonicated in dichloromethane for 20 min prior to drying with air.

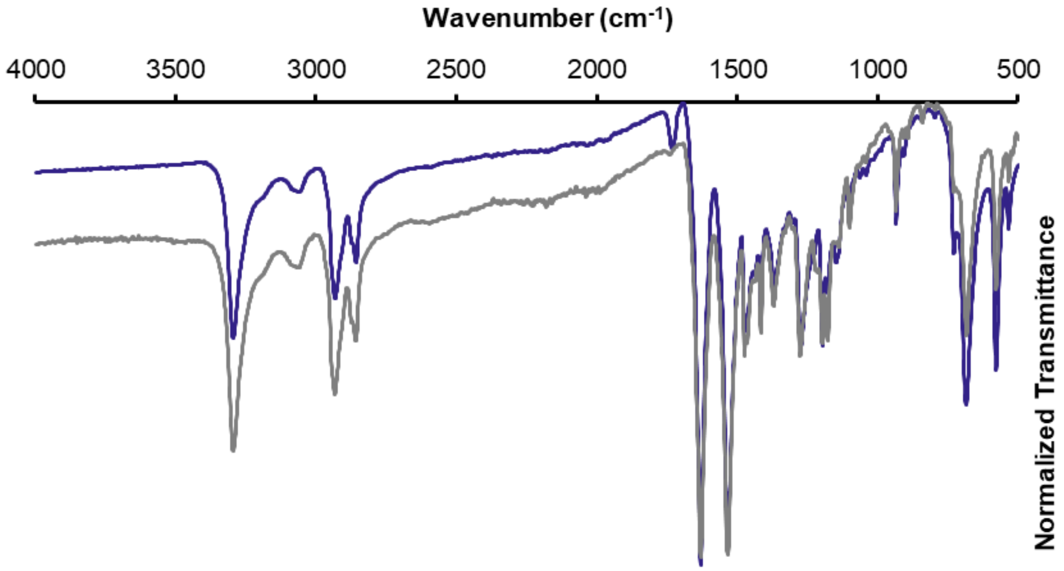

Figure S8. FT-IR spectra of nylon-6,6 before (gray) and after (purple) drop-casting with P*t*BA.

PET-RAFT in the presence of nylon-6,6

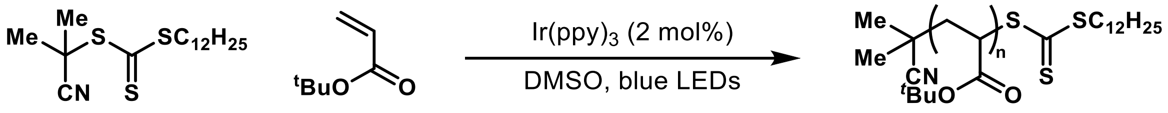

Poly(*tert*-butyl acrylate) (P^{t}BA) samples in the presence of nylon-6,6 were prepared according to published PET-RAFT conditions. Ir(ppy)$_3$ (1.0 mg, 2.7 x 10^{-4} equiv, 0.015 µmol), 2-cyano-2-propyl dodecyl trithiocarbonate (19 mg, 1 equiv, 0.055 mmol), *tert*-butyl acrylate (0.81 mL, 100 equiv, 5.5 mmol), a solvent-cast nylon-6,6 film, and DMSO (0.6 mL) were combined in a one-dram vial. The solution was sparged with nitrogen and placed in a blue LED dish. The sample was irradiated for three hours with cooling, after which the reaction was opened to air and aliquots were taken for NMR and GPC analysis. Volatiles were removed in vacuo and the resulting polymer was purified by precipitating three times in cold methanol. ^{1}H NMR: 78% conversion, M_n^{theo} = 10 kg/mol. GPC: M_n^{RI}=2.7 kg/mol, *Đ*=1.85. The nylon-6,6 substrate was sonicated dichloromethane for 20 min prior to drying under ambient conditions.

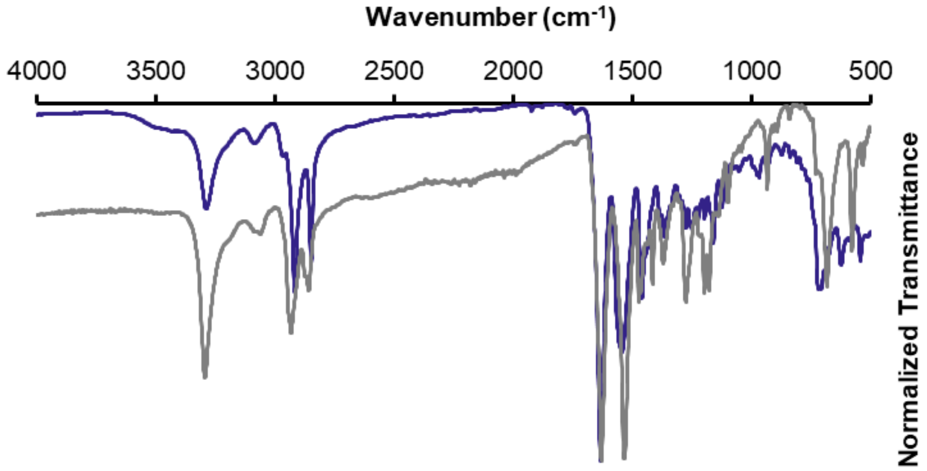

Figure S9. FT-IR spectra of nylon-6,6 before (gray) and after (purple) PET-RAFT with P^{t}BA.

Standard conditions, no HAT catalyst

In a nitrogen-filled glovebox, BisTTC (1 equiv, 0.01 mmol, 5.5 mg), *tert*-butyl acrylate (200 equiv, 2 mmol, 0.29 mL), and dioxane (0.25 mL) were combined in a one-dram vial. The solution was syringed onto a flat nylon-6,6 substrate, which was placed on a glass microscope slide. The reaction mixture was covered with a second glass microscope slide and irradiated with a CFL lamp placed 4 cm away without air cooling. The reaction was allowed to proceed for 18 hours before the light was turned off and the substrate removed from the glovebox. The substrate was sonicated in dichloromethane for 20 min prior to drying with air.

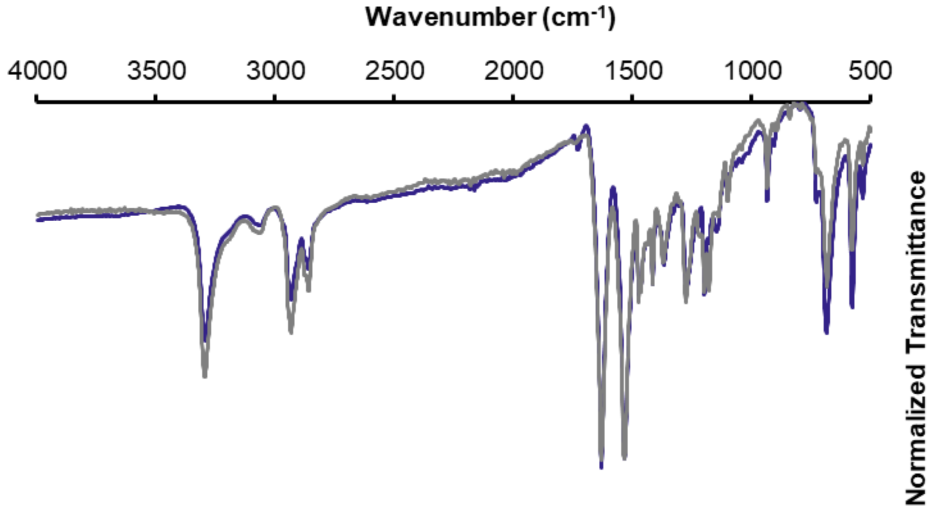

Figure S10. FT-IR spectra of nylon-6,6 before (gray) and after (purple) standard reaction conditions with thioxanthone as a photocatalyst.

Standard conditions, no light

In a nitrogen-filled glovebox, 2,2'diOMeTX (1 equiv, 0.01 mmol, 2.7 mg), BisTTC (1 equiv, 0.01 mmol, 5.5 mg), and *tert*-butyl acrylate (200 equiv, 2 mmol, 0.29 mL), and dioxane (0.25 mL) were combined in a one-dram vial. The solution was syringed onto a flat nylon-6,6 substrate, which was laid on a glass microscope slide. The reaction mixture was covered with a second glass microscope slide and covered with aluminum foil, where it sat for 18 hours in the dark before the substrate was removed from the glovebox. The substrate was sonicated in dichloromethane for 20 min prior to drying with air.

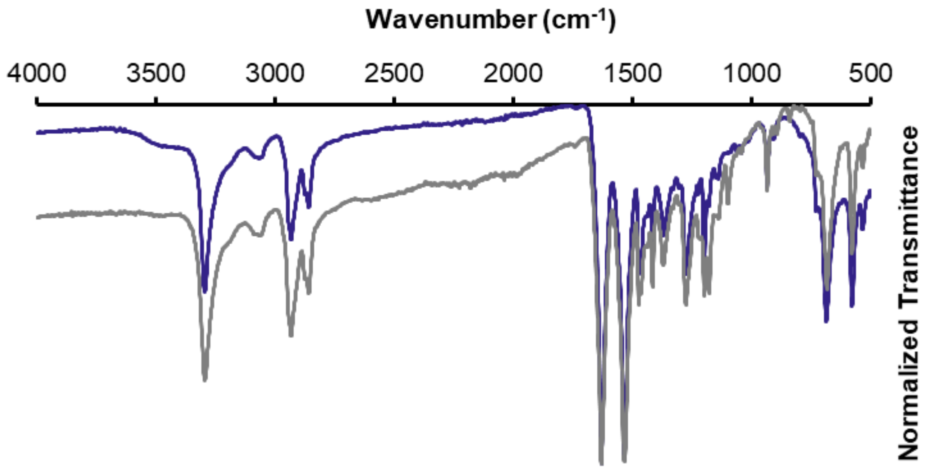

Figure S11. FT-IR spectra of nylon-6,6 before (gray) and after (purple) standard reaction conditions with no light.

Standard conditions, no bis(trithiocarbonate) disulfide

In a nitrogen-filled glovebox, 2,2'diOMeTX (1 equiv, 0.01 mmol, 2.7 mg), *tert*-butyl acrylate (200 equiv, 2 mmol, 0.29 mL), and dioxane (0.25 mL) were combined in a one-dram vial. The solution was syringed onto a flat nylon-6,6 substrate, which was placed on a glass microscope slide. The reaction mixture was covered with a second glass microscope slide and irradiated with a CFL lamp placed 4 cm away without air cooling. The reaction was allowed to proceed for 18 hours before the light was turned off and the substrate removed from the glovebox. The substrate was sonicated in dichloromethane for 20 min prior to drying with air.

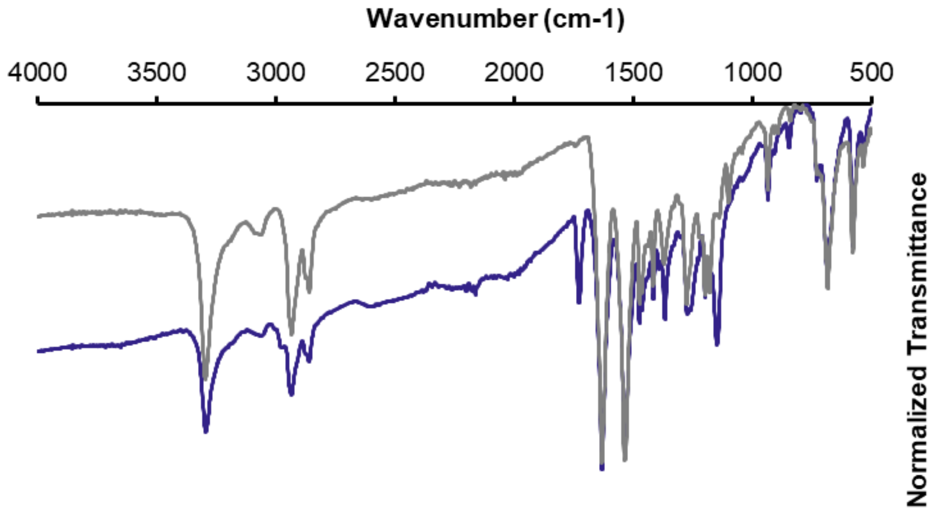

Figure S12. FT-IR spectra of nylon-6,6 before (gray) and after (purple) standard reaction conditions with no disulfide.

Solvent screen under standard conditions

In a nitrogen-filled glovebox, 2,2'diOMeTX (1 equiv, 0.01 mmol, 2.7 mg), BisTTC (1 equiv, 0.01 mmol, 5.5 mg), and *tert*-butyl acrylate (200 equiv, 2 mmol, 0.29 mL), and solvent (0.25 mL) were combined in a one-dram vial. The solution was syringed onto a flat nylon-6,6 substrate, which was laid on a glass microscope slide. The reaction mixture was covered with a second glass microscope slide and irradiated with a CFL lamp placed 4 cm away without air cooling. The reaction was allowed to proceed for 18 hours before the light was turned off and the substrate removed from the glovebox. The substrate was sonicated in dichloromethane for 20 min prior to drying with air.

Solvent	R_A
**Dioxane (standard conditions)**	1.01
**Toluene**	0.48
**Cyclohexane**	0.23
**DCE**	0.16
**THF**	0.65

Table S1. R_A of resulting P^{t}BA stretches under standard conditions with various solvents.

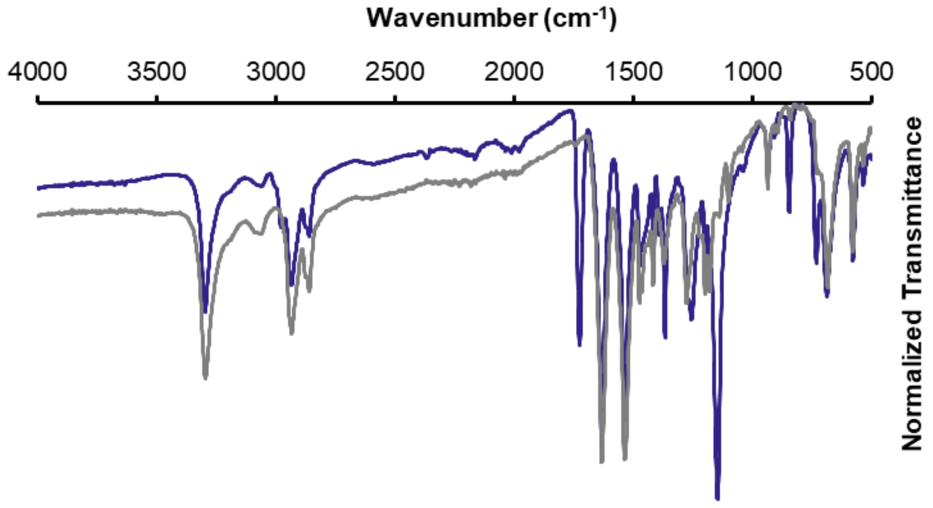

Figure S13. FT-IR spectra of nylon-6,6 before (gray) and after (purple) standard reaction conditions with toluene.

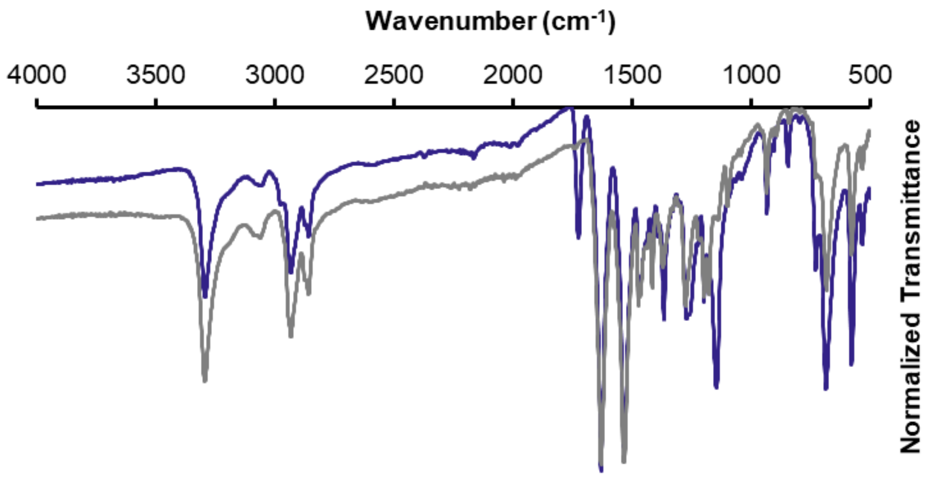

Figure S14. FT-IR spectra of nylon-6,6 before (gray) and after (purple) standard reaction conditions with cyclohexane.

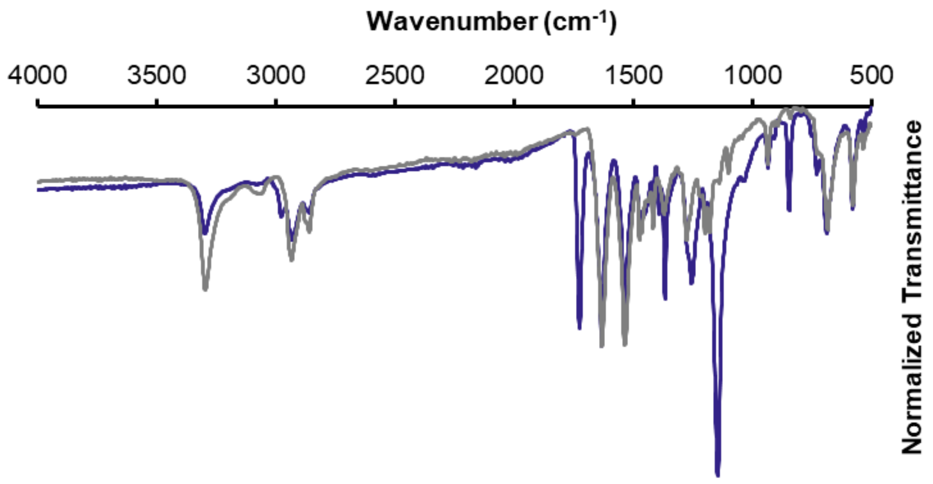

Figure S15. FT-IR spectra of nylon-6,6 before (gray) and after (purple) standard reaction conditions with THF.

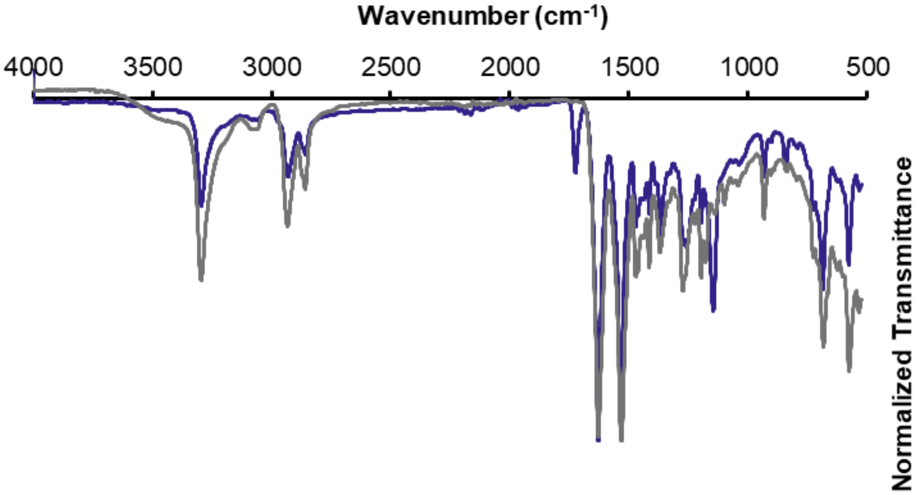

Figure S16. FT-IR spectra of nylon-6,6 before (gray) and after (purple) standard reaction conditions with DCE.

Standard conditions for a chain extension

In a nitrogen-filled glovebox, 2,2'diOMeTX (1 equiv, 0.01 mmol, 2.7 mg), BisTTC (1 equiv, 0.01 mmol, 5.5 mg), *tert*-butyl acrylate (200 equiv, 2 mmol, 0.29 mL), and dioxane (0.25 mL) were combined in a one-dram vial. The solution was syringed onto a flat nylon-6,6 substrate, which was placed on a glass microscope slide. The reaction mixture was covered with a second glass microscope slide and irradiated with a CFL lamp placed 4 cm away without air cooling. The reaction was allowed to proceed for 3 hours before the light was turned off and the substrate removed from the glovebox. The substrate was sonicated in dichloromethane for 20 min prior to drying with air. The sample was then added to a scintillation vial with AIBN (0.05 equiv, 6 µmol, 1 mg), methyl acrylate (200 equiv, 12 mmol, 1.08 mL), and toluene (3 mL) and freeze-pump thawed a total of three times before backfilling with nitrogen. The reaction was heated at 70 °C for 18 hours. The substrate was sonicated in dichloromethane for 20 min prior to drying with air.

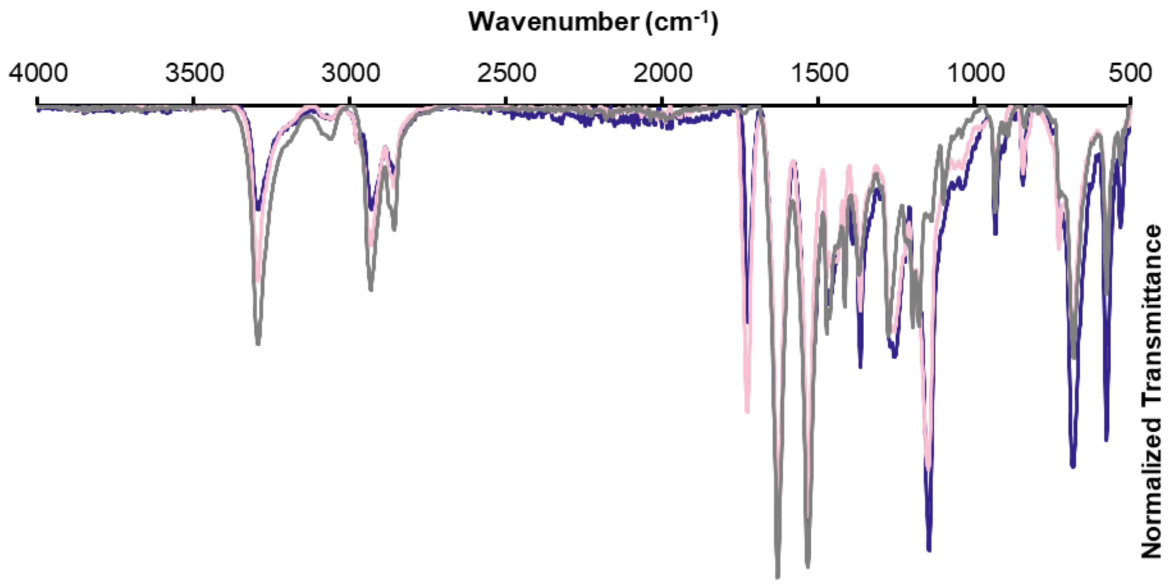

Figure S17. FT-IR spectra of nylon-6,6 before (gray) and after (purple) standard reaction conditions for 3 hours. Subsequent chain extension with methyl acrylate is shown in pink.

S8. Synthesis of brush polymers for XPS and AFM studies

Preparation of nylon-6,6,-g-P^{t}BuA on a silicon wafer

In a nitrogen-filled glovebox, 2,2'diOMeTX (1 equiv, 0.01 mmol), BisTTC (1 equiv, 0.01 mmol), *tert*-butyl acrylate (200 equiv, 2 mmol), and solvent (0.25 mL) were combined in a one-dram vial. The solution was syringed onto a silicon wafer spin-coated with nylon-6,6, which was placed on a glass microscope slide. The reaction mixture was covered with a second glass microscope slide and irradiated with a CFL lamp placed 4 cm away without air cooling. The reaction was allowed to proceed for 18 hours before the light was turned off and the substrate removed from the glovebox. The substrate was washed with dichloromethane prior to drying under ambient conditions.

S9. XPS studies

Samples were analyzed using a Thermo Scientific Nexsa G2Spectrometer with operating pressure ca. 1x10-9 Torr. Monochromatic Al Kα x rays (1486.6 eV) with photoelectrons collected from a 400 µm diameter analysis spot. Photoelectrons were collected at a 90° emission angle with source to analyzer angle of 54.7°. A hemispherical analyzer determined electron kinetic energy, using a pass energy of 200 eV for wide/survey scans, and 50 eV for high resolution scans. A flood gun was used for charge neutralization of non-conductive samples.

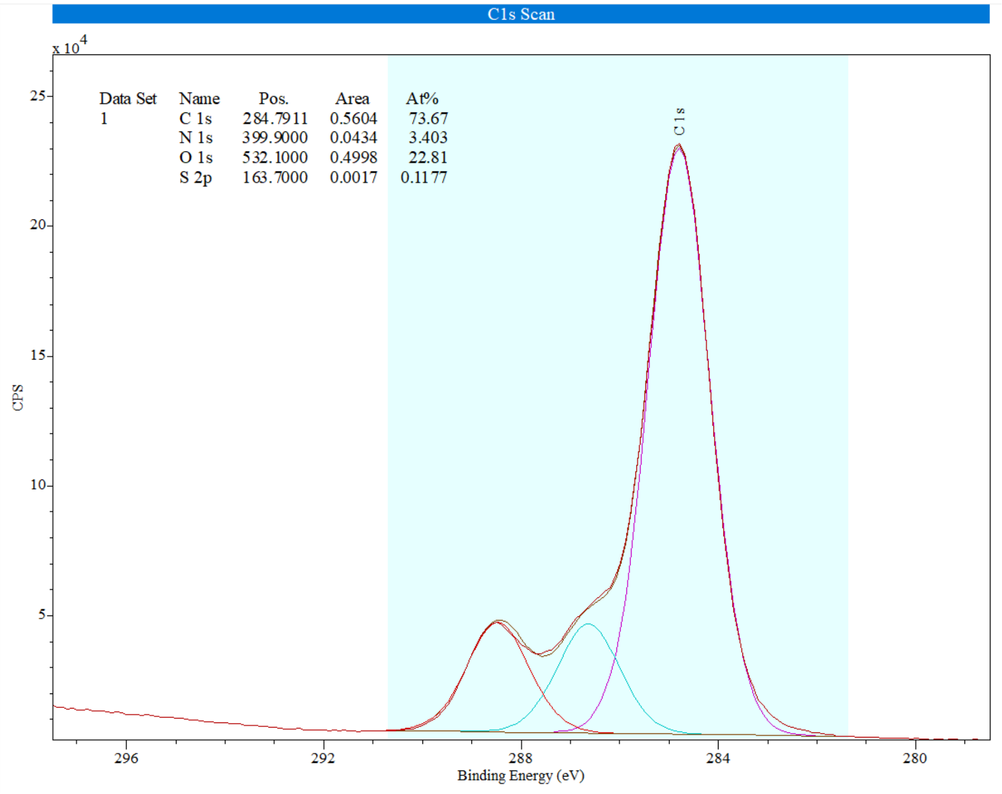

Figure S18. XPS Spectrum of nylon-6,6-g-P^{t}BA, carbon high resolution scan.

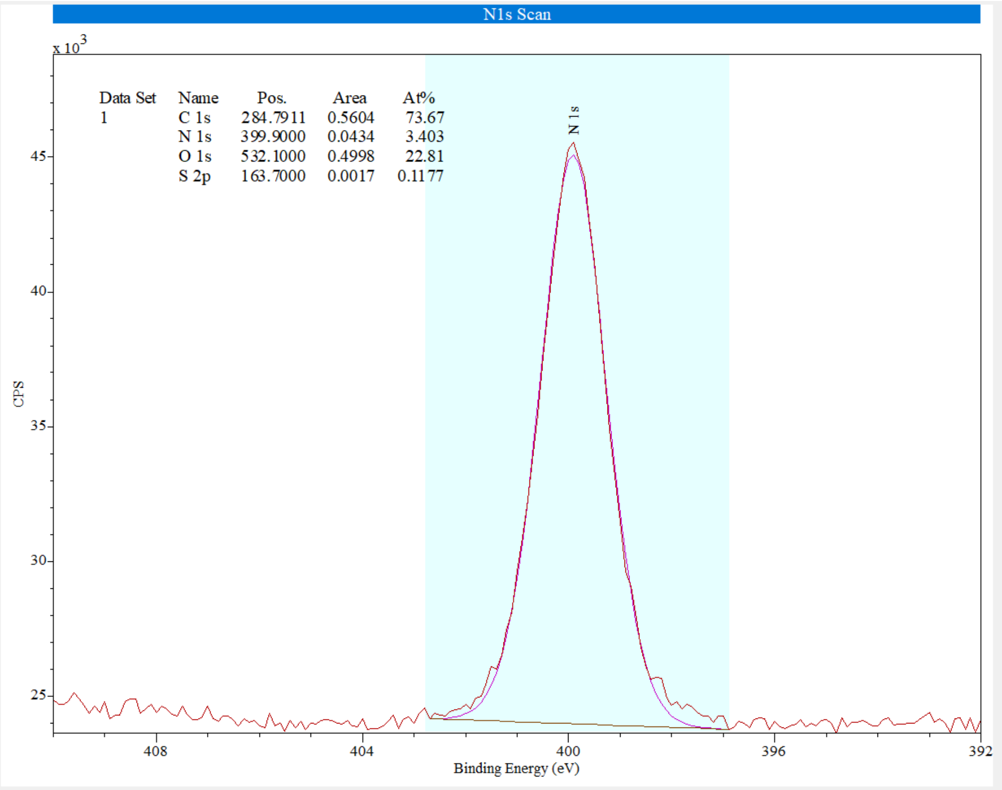

Figure S19. XPS Spectrum of nylon-6,6-g-P^{t}BA, nitrogen high resolution scan.

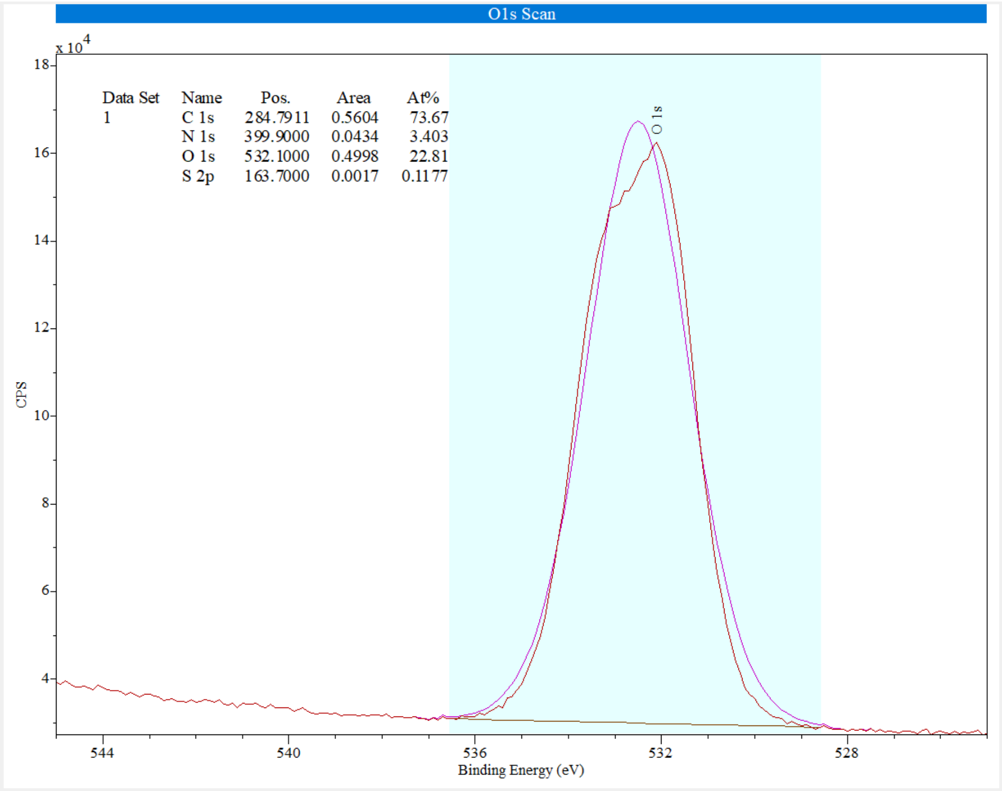

Figure S20. XPS spectrum of nylon-6,6-g-P^{t}BA, oxygen high resolution scan.

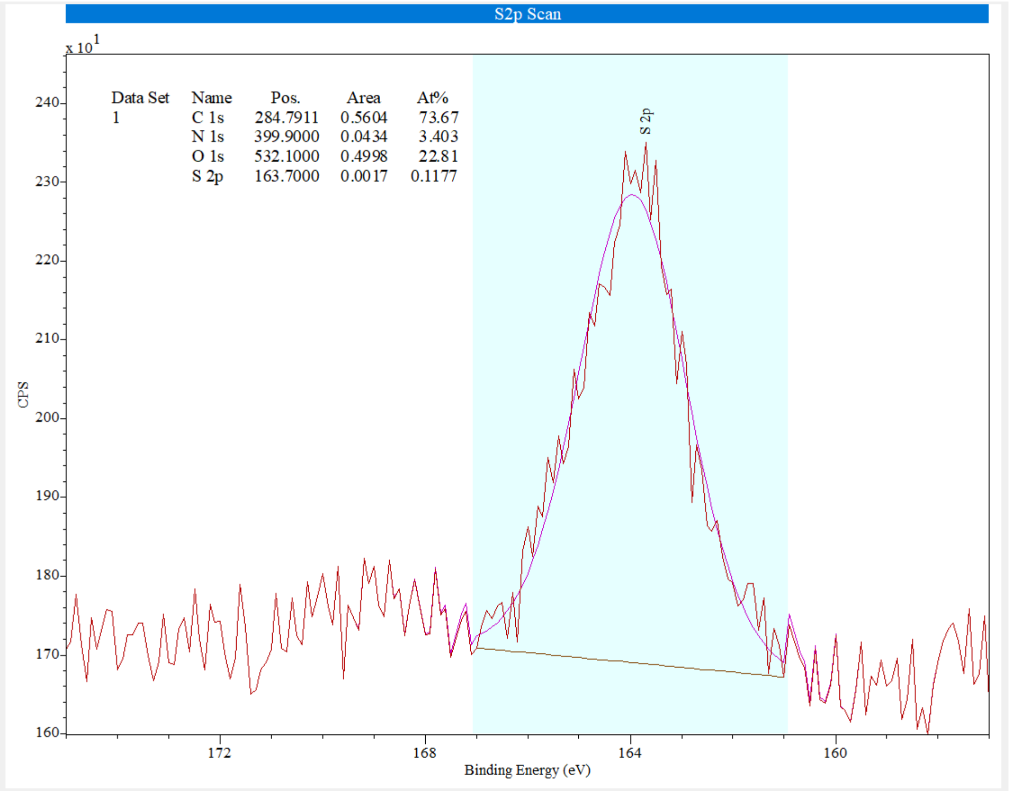

Figure S21. XPS spectrum of nylon-6,6-g-P^{t}BA, sulfur high resolution scan.

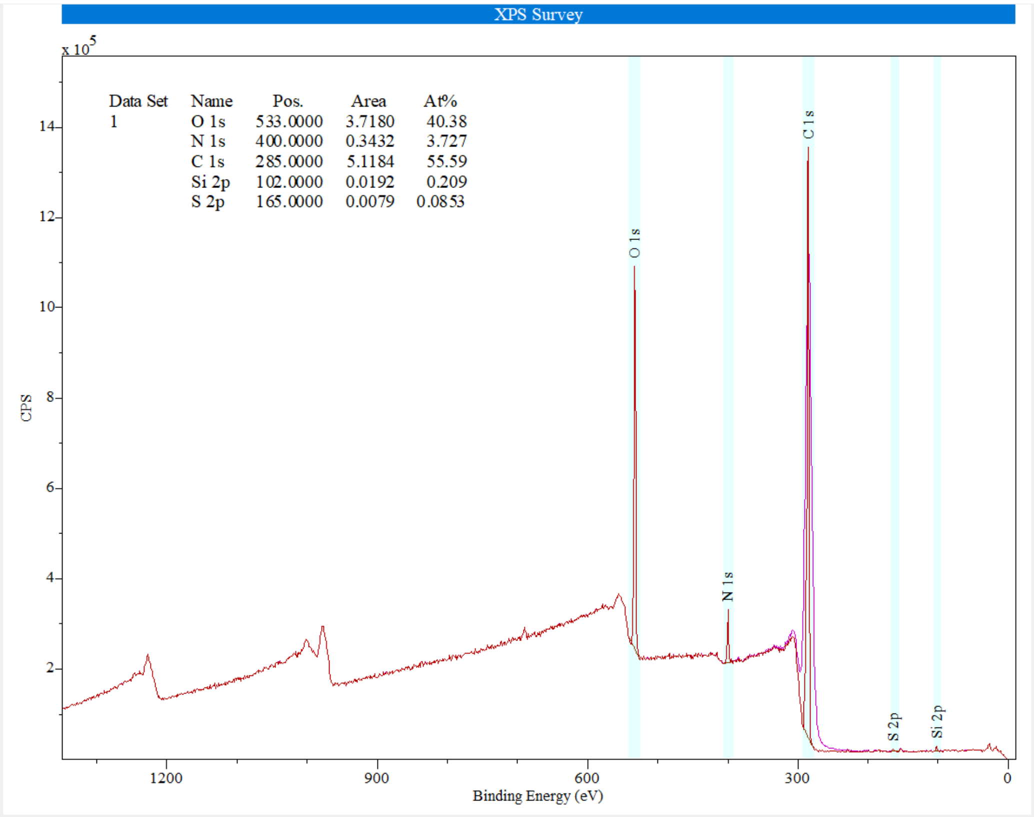

Figure S22. XPS spectrum of nylon-6,6-g-P^{t}BA, survey scan.

S10. Discussion of proposed SI HAT-RAFT mechanism using 2,2'diOMeTX as a hydrogen atom abstraction catalyst

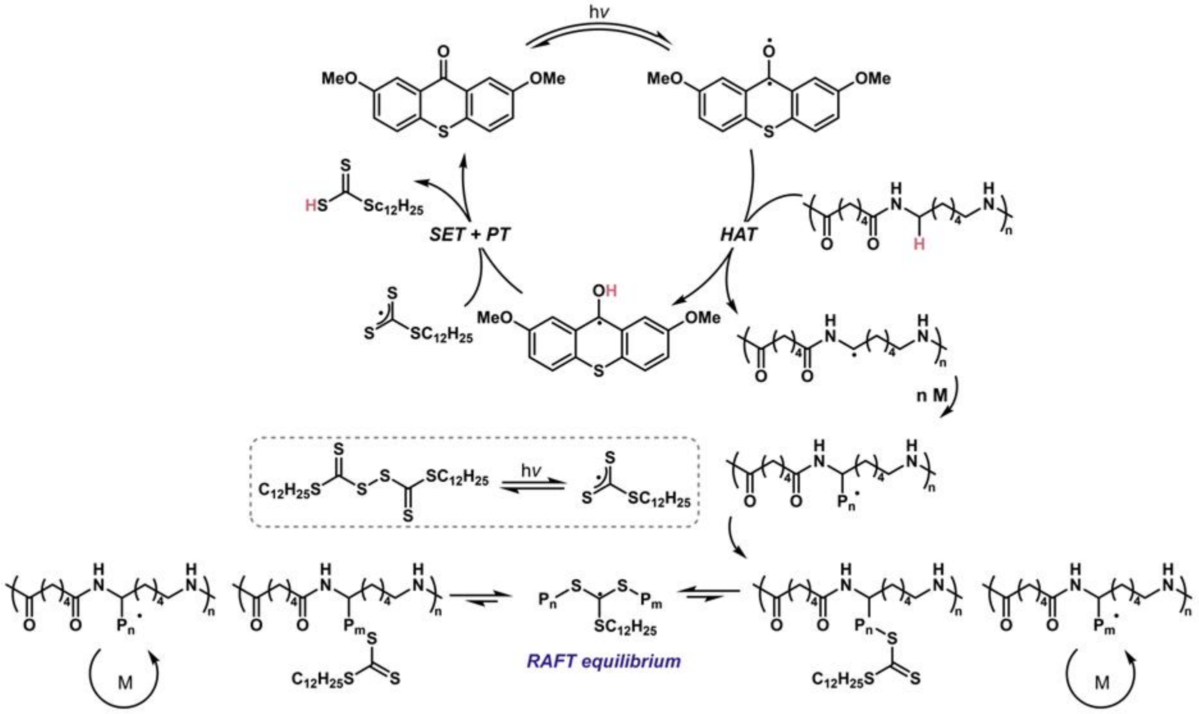

Figure S23. Proposed SI HAT-RAFT mechanism.

Catalyst selectivity

Figure S22 depicts hydrogen abstraction from the carbon α to the amide in the nylon-6,6 repeat unit. As is discussed in the main text, small-molecule studies have reported a high degree of selectivity for abstraction from hydridic C-H positions by alkoxy radicals. In the case of aliphatic amides, this is the site alpha to the nitrogen.[10–12] We propose that the kinetic selectivity of the catalyst for abstracting from these hydridic C-H bonds precludes unwanted radical generation on the polymer backbone. This is supported by the fact that we observe only linear polymer chains when initiating polymerization from small-molecules in solution.[3] While studies by Ruehe and coworkers have shown that benzophenone-functionalized polymers can abstract a hydrogen from (meth)acrylic polymer samples to form a crosslinked network via C,H-insertion,[13,14] they also report that free benzophenone is less likely to initiate crosslinking compared to pendent-benzophenone, as the only mechanism for crosslinking would be coupling of aliphatic radicals, which is not likely to occur in (meth)acrylic polymer samples.[15] The presence of polarity-matched hydrogen donors (such as the polyamide surface or dioxane) and monomer in solution impedes hydrogen abstraction from the polymer backbone. To confirm that linear polymer brushes were being formed on the surface, as opposed to a cross-linked network resulting from non-selective hydrogen abstraction, we tested the solubility of nylon-6,6-*g*-P^{t}BA in HFIP. Figure S23 illustrates that a nylon-6,6-*g*-P^{t}BA sample could be fully dissolved in HFIP, indicating no crosslinking.

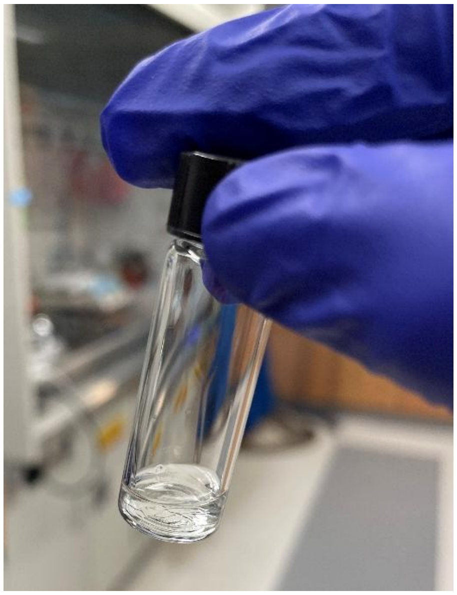 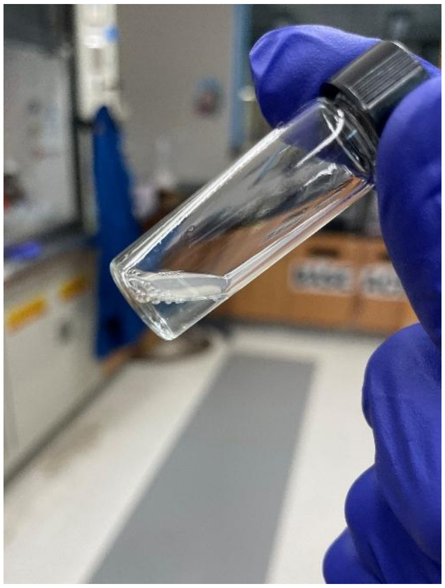

Figure S24. Nylon-6,6-*g*-P^{t}BA immediately after dissolving in HFIP (left) and 5 min after dissolving (right).

Catalyst turnover

Removal of BisTTC results in inhibited grafting by FT-IR (Figure 1, main text), indicating that BisTTC is necessary for efficient grafting. In our previous report on small-molecule initiated HAT-RAFT, we directly observe the formation of trithiocarbonic acid, the product of disulfide reduction by photocatalyst, via MALDI mass-spectrometry.[3] Additionally, ketyl radicals readily undergo single-electron transfer events in the presence of an oxidant.[11] Based on the reducing nature of ketyl radicals and evidence that trithiocarbonic acid is being formed, catalyst turnover is theorized to proceed via single electron transfer and proton transfer from the thioxanthone ketyl radical to the trithiocarbonyl radical to generate trithiocarbonic acid.

S11. Brush Height Analysis

Brush height measurements by AFM

A silicon wafer spin-coated with nylon-6,6 was scratched with a razor blade and step-height measurements were taken at the boundary between scratched and unscratched regions. The sample was then subject to reaction conditions as described above. The depth of the same scratch was measured after brush growth, and the average distance between the two measurements in reported as the brush height.

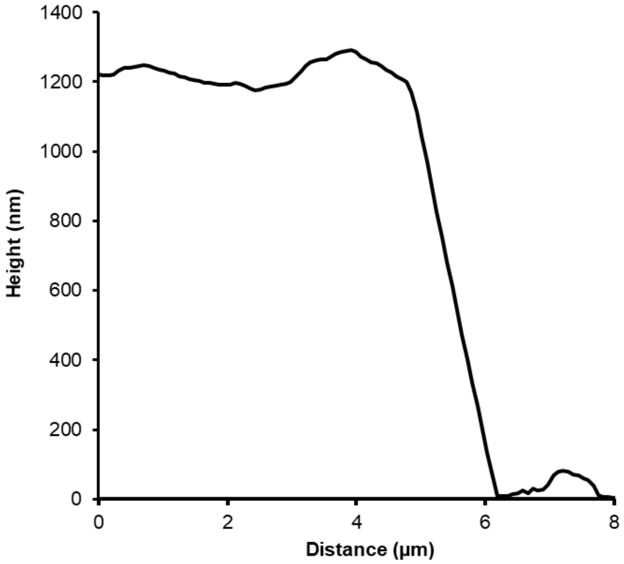

Figure S25. AFM profile of boundary between scratched and unscratched regions of an unfunctionalized nylon-6,6 film.

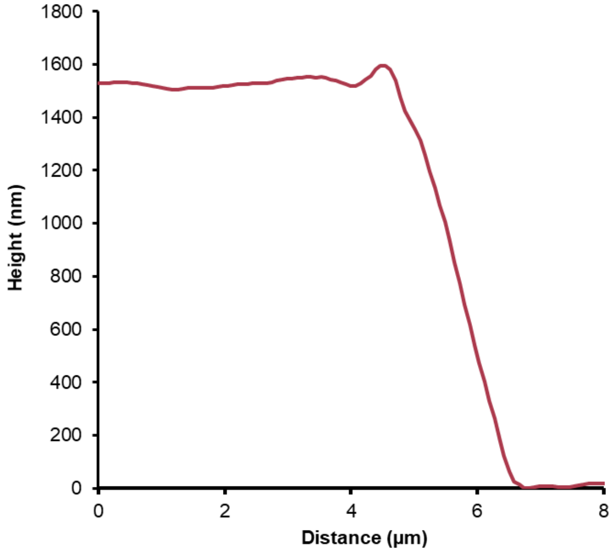

Figure S26. AFM profile of boundary between scratched and unscratched regions of a nylon-6,6-*g*-P^{t}BA film.

Brush height measurements by optical profilometry (film subject to standard reaction conditions with 2,2'diOMeTX)

A silicon wafer spin-coated with nylon-6,6 was scratched with a razor blade and step-height measurements at 50x magnification were taken at the boundary between scratched and unscratched regions. The sample was then subject to standard reaction conditions with 2,2'diOMeTX as described above. The depth of the same scratch was measured after brush growth, and the average distance between the two measurements in reported as the brush height. The brush thickness under standard conditions with 2,2'diOMeTX was measured at 300 nm

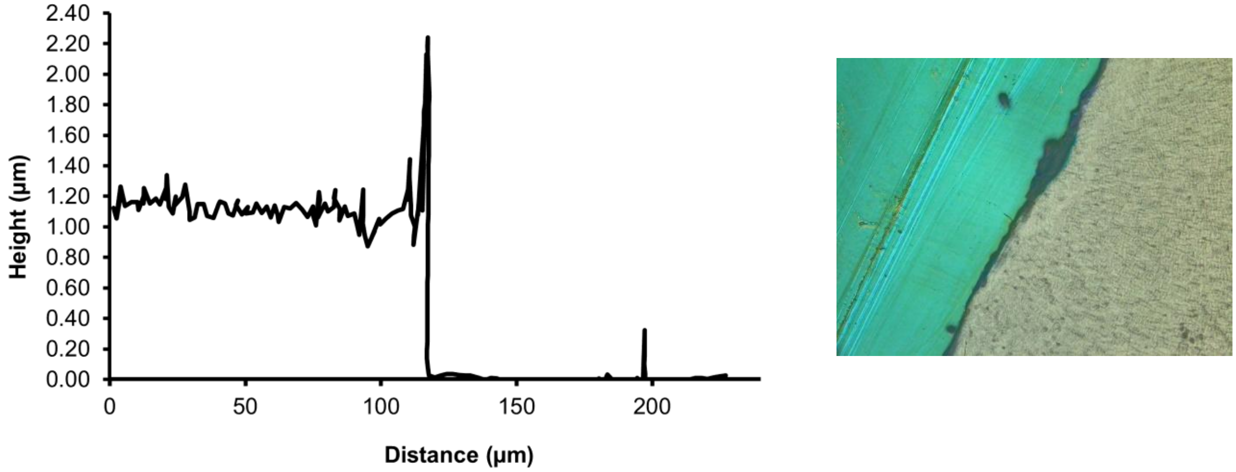

Figure S27. A. Height profile of boundary between scratched and unscratched regions of an unfunctionalized nylon-6,6 film. B. Profilometer image of unfunctionalized nylon-6,6 film. The average scratch depth was measured as 986 nm.

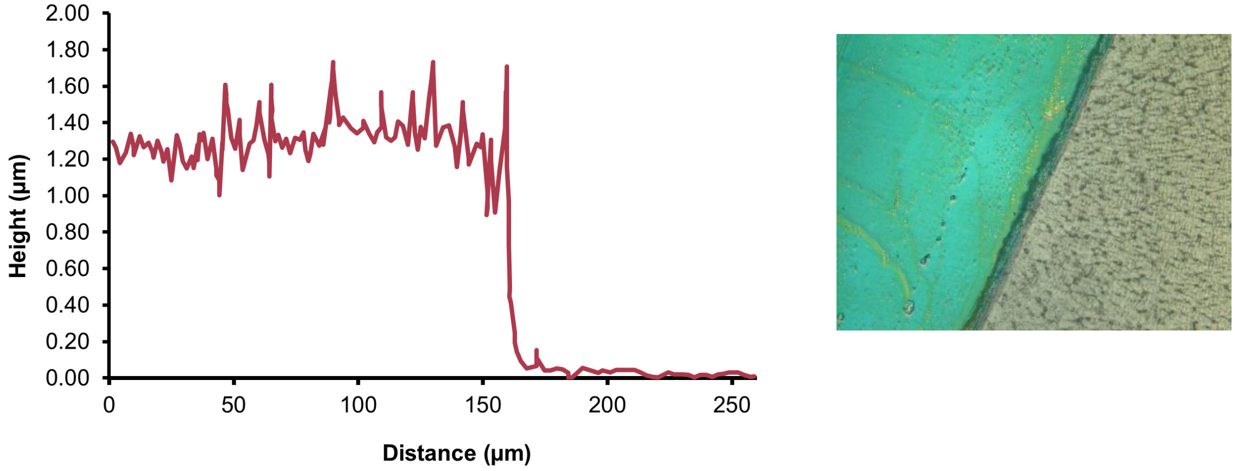

Figure S28. A. Height profile of boundary between scratched and unscratched regions of a nylon-6,6-*g*-P^{t}BA film. B. Profilometer image of the nylon-6,6-*g*-P^{t}BA film. The average scratch depth was measured as 1285 nm.

Probing the effect of pre-swelling on polymerization

In a nitrogen-filled glovebox, 2,2'diOMeTX (1 equiv, 0.01 mmol, 2.7 mg), BisTTC (1 equiv, 0.01 mmol, 5.5 mg), *tert*-butyl acrylate (200 equiv, 2 mmol, 0.29 mL), and dioxane (0.25 mL) were combined in a one-dram vial. A nylon-6,6 substrate was submerged in the solution for 14 hours prior to placing the substrate on a glass microscope slide. The film was covered with a second glass microscope slide and irradiated with a CFL lamp placed 4 cm away without air cooling. The reaction was allowed to proceed for 7 hours before the light was turned off and the substrate removed from the glovebox. The substrate was sonicated in dichloromethane for 20 min prior to drying with air.

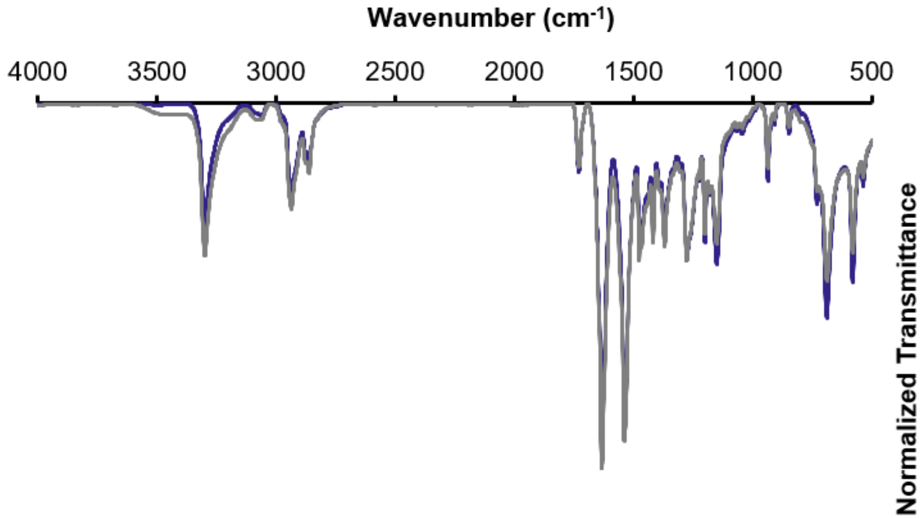

Figure S29. FT-IR spectra of nylon-6,6-*g*-P^{t}BA polymerized under standard conditions for 7 hours (gray) and nylon-6,6-*g*-P^{t}BA polymerized by pre-swelling the substrate (purple).

Under our standard conditions, the nylon substrate is sandwiched between glass-slides with the polymerization mixture during irradiation. The polymerization mixture is likely maximally swelling the substrate during this time. This would result in minimal difference between the pre-swollen substrate and standard reaction conditions. The effect of swelling time on grafting efficiency in SI HAT-RAFT warrants further investigation.

Brush height measurements by optical profilometry (film subject to standard reaction conditions with TX)

A silicon wafer spin-coated with nylon-6,6 was scratched with a razor blade and step-height measurements at 50x magnification were taken at the boundary between scratched and unscratched regions. The sample was then subject to standard reaction conditions with TX as described above. The depth of the same scratch was measured after brush growth, and the average distance between the two measurements in reported as the brush height. The brush thickness under standard conditions with TX was measured at 130 nm.

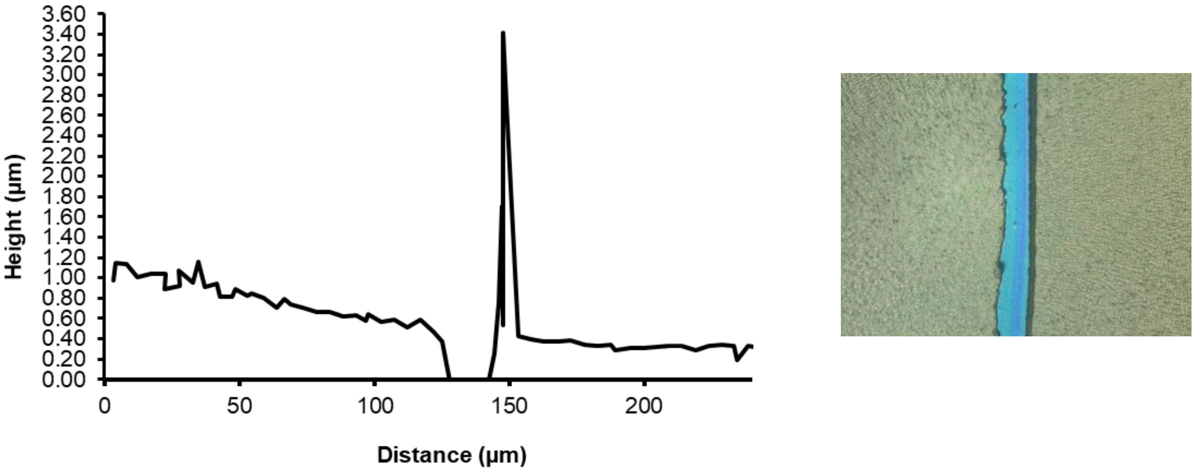

Figure S30. A. Height profile of boundary between scratched and unscratched regions of an unfunctionalized nylon-6,6 film. B. Profilometer image of unfunctionalized nylon-6,6 film. The average scratch depth was measured as 463 nm.

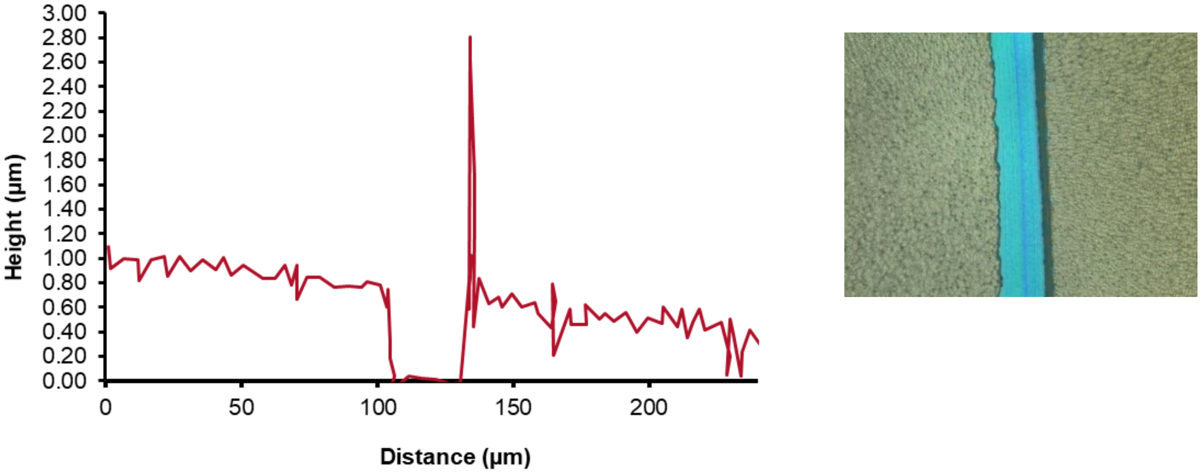

Figure S31. A. Height profile of boundary between scratched and unscratched regions of a nylon-6,6-*g*-P^{t}BA film. B. Profilometer image of the nylon-6,6-*g*-P^{t}BA film. The average scratch depth was measured as 592 nm.

Brush height measurements by optical profilometry (film subject to standard reaction conditions with MTBP)

A silicon wafer spin-coated with nylon-6,6 was scratched with a razor blade and step-height measurements at 50x magnification were taken at the boundary between scratched and unscratched regions. The sample was then subject to standard reaction conditions with MTBP as described above. The depth of the same scratch was measured after brush growth, and the average distance between the two measurements in reported as the brush height. The brush thickness under standard conditions with MTBP was measured at 74 nm.

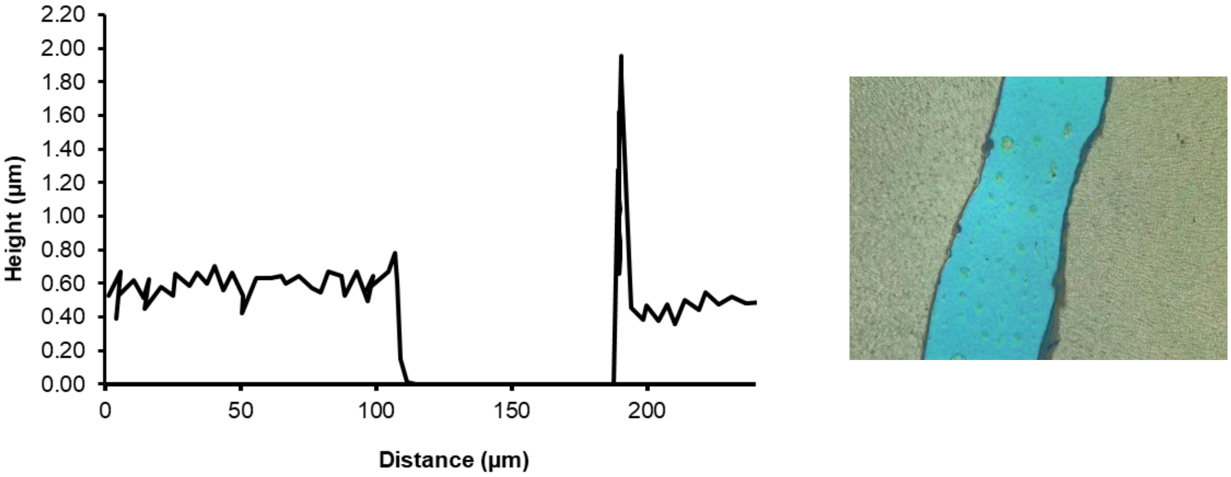

Figure S32. A. Height profile of boundary between scratched and unscratched regions of an unfunctionalized nylon-6,6 film. B. Profilometer image of unfunctionalized nylon-6,6 film. The average scratch depth was measured as 681 nm.

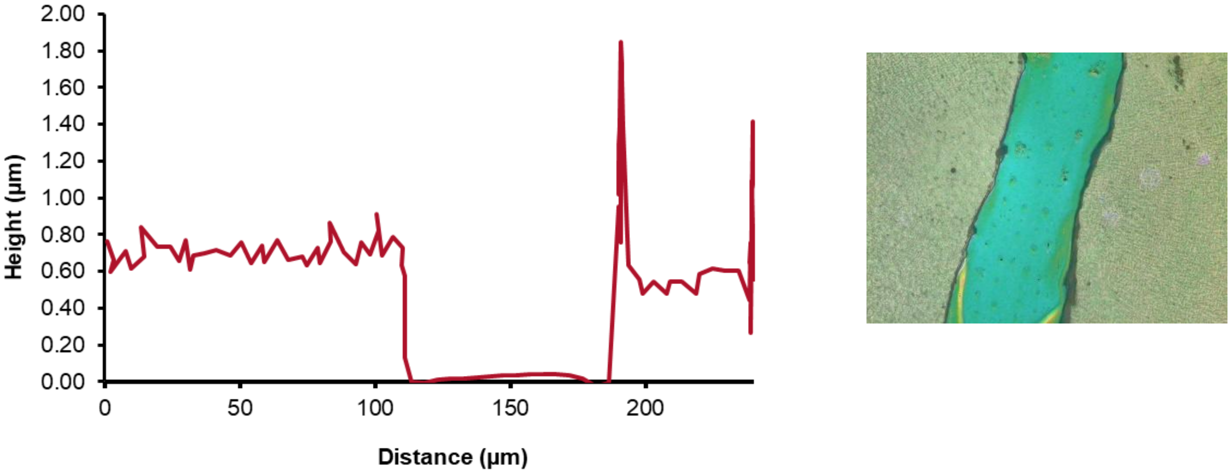

Figure S33. A. Height profile of boundary between scratched and unscratched regions of a nylon-6,6-*g*-P^{t}BA film. B. Profilometer image of the nylon-6,6-*g*-P^{t}BA film. The average scratch depth was measured as 755 nm.

Brush height measurements by optical profilometry (film subject to standard reaction conditions with 2,2'diOMeTX, then a thermal RAFT chain extension)

A silicon wafer spin-coated with nylon-6,6 was scratched with a razor blade and step-height measurements at 50x magnification were taken at the boundary between scratched and unscratched regions. The sample was then subject to standard reaction conditions with 2,2'diOMeTX and ^{t}BA for 2 h. The depth of the same scratch was measured after brush growth, and the average distance between the two measurements in reported as the brush height. The brush thickness after 2 h of irradiation was 33 nm. The same sample was then subjected to thermal RAFT chain extension conditions with MA, as described above. Brush thickness increased by 55 nm after subjecting the sample to chain extension conditions, for a total brush thickness of ~90 nm.

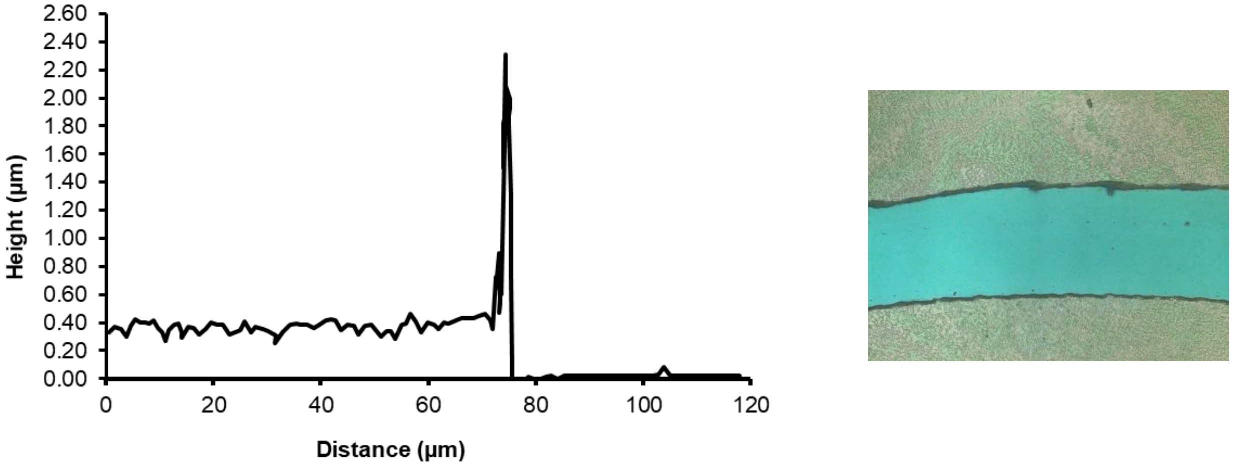

Figure S34. A. Height profile of boundary between scratched and unscratched regions of an unfunctionalized nylon-6,6 film. B. Profilometer image of unfunctionalized nylon-6,6 film. The average scratch depth was measured as 337 nm.

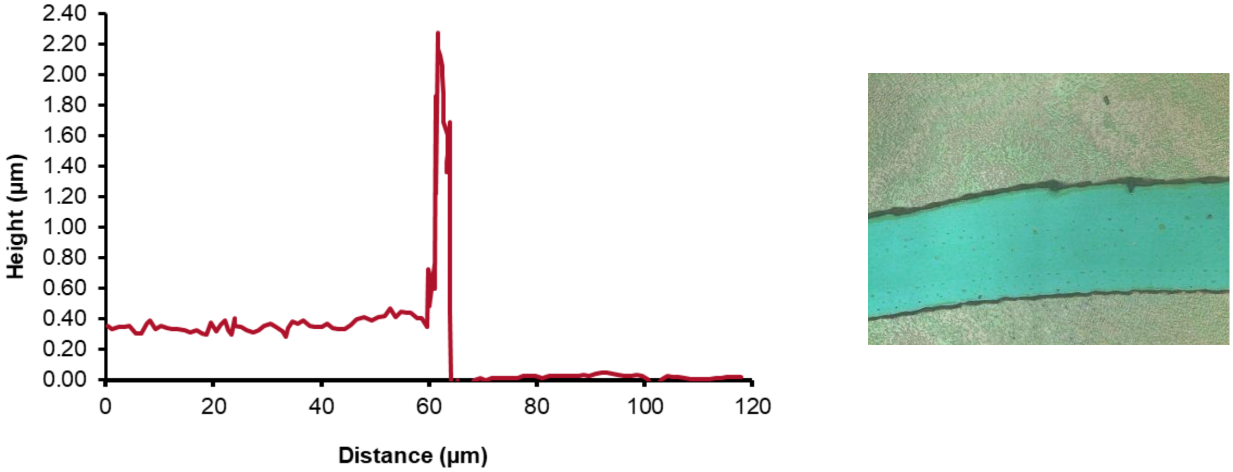

Figure S35. A. Height profile of boundary between scratched and unscratched regions of a nylon-6,6-*g*-P^{t}BA film. B. Profilometer image of the nylon-6,6-*g*-P^{t}BA film. The average scratch depth was measured as 370 nm.

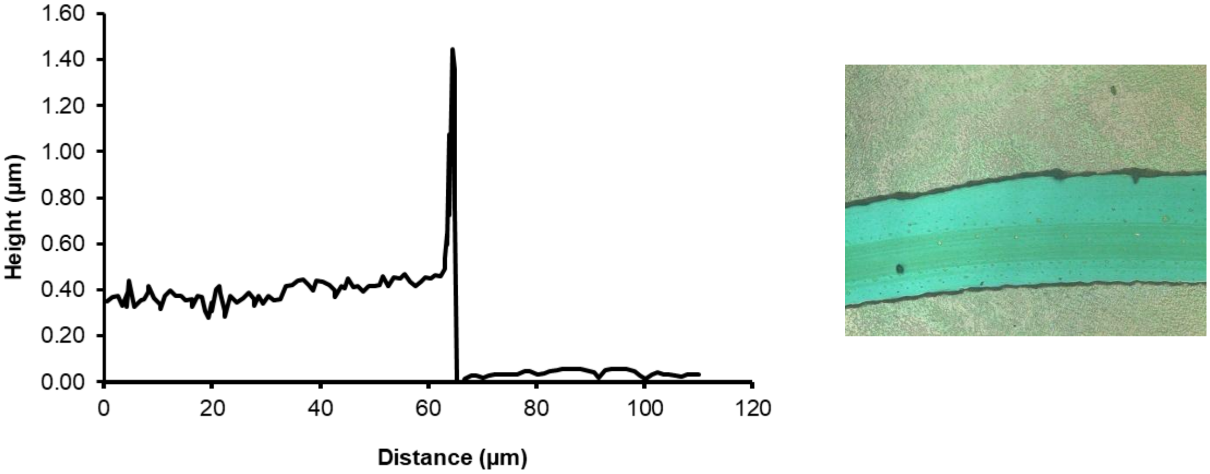

Figure S36. A. Height profile of boundary between scratched and unscratched regions of a nylon-6,6-*g*-P^{t}BA film. B. Profilometer image of the nylon-6,6-*g*-P^{t}BA film. The average scratch depth was measured as 425 nm.

S12. Synthesis of Brush Polymers for Monomer Scope

Synthesis of nylon-6,6-g-P^{t}BA

In a nitrogen-filled glovebox, 2,2'diOMeTX (1 equiv, 0.01 mmol, 2.7 mg), BisTTC (1 equiv, 0.01 mmol, 5.5 mg), *tert*-butyl acrylate (200 equiv, 2 mmol, 0.29 mL), and dioxane (0.25 mL) were combined in a one-dram vial. The solution was syringed onto a flat-solvent cast nylon-6,6 substrate, which was placed on a glass microscope slide. The reaction mixture was covered with a second glass microscope slide and irradiated with a CFL lamp placed 4 cm away without air cooling. The reaction was allowed to proceed for 18 hours before the light was turned off and the substrate removed from the glovebox. The substrate was sonicated in dichloromethane for 20 min prior to drying under ambient conditions.

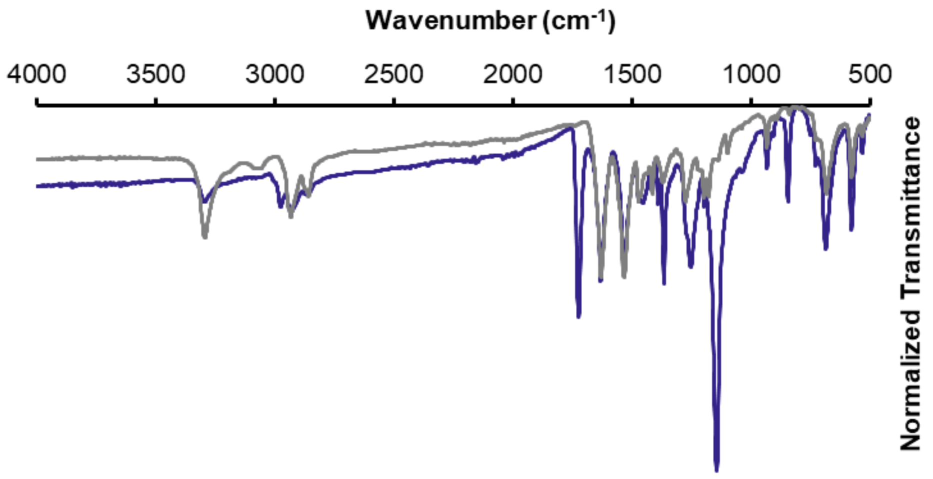

Figure S37. FT-IR spectra of nylon-6,6 before (gray) and after (purple) standard reaction conditions with *tert*-butyl acrylate.

Synthesis of nylon-6,6-g-PTMSPMA

In a nitrogen-filled glovebox, 2,2'diOMeTX (1 equiv, 0.01 mmol, 2.7 mg), BisTTC (1 equiv, 0.01 mmol, 5.5 mg), 3-(trimethoxysilyl)propyl methacrylate (200 equiv, 2 mmol, 0.48 mL), and dioxane (0.25 mL) were combined in a one-dram vial. The solution was syringed onto a flat-solvent cast nylon-6,6 substrate, which was placed on a glass microscope slide. The reaction mixture was covered with a second glass microscope slide and irradiated with a CFL lamp placed 2 cm away without air cooling. The reaction was allowed to proceed for 18 hours before the light was turned off and the substrate removed from the glovebox. The substrate was sonicated in dichloromethane for 20 min prior to drying under ambient conditions.

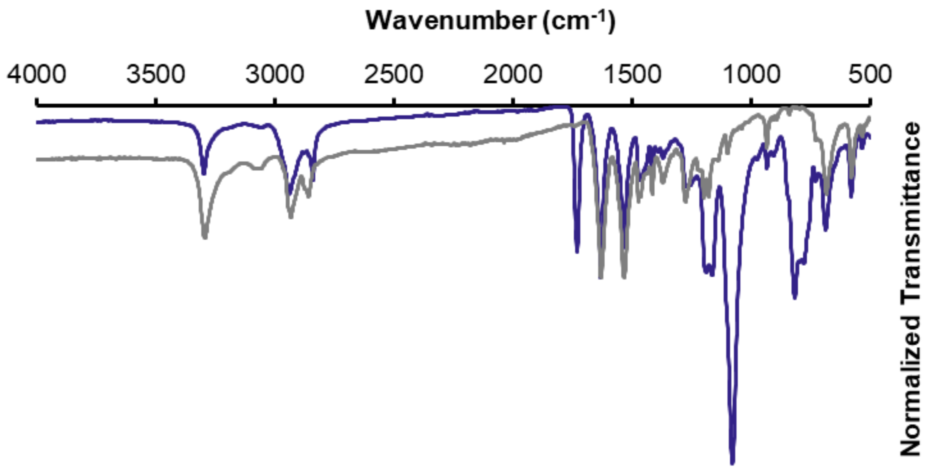

Figure S38. FT-IR spectra of nylon-6,6 before (gray) and after (purple) standard reaction conditions with TMSPA.

Synthesis of nylon-6,6-g-PHEMA

In a nitrogen-filled glovebox, 2,2'diOMeTX (1 equiv, 0.01 mmol, 2.7 mg), BisTTC (1 equiv, 0.01 mmol, 5.5 mg), hydroxyethyl methacrylate (200 equiv, 2 mmol, 0.24 mL), and dioxane (0.25 mL) were combined in a one-dram vial. The solution was syringed onto a flat-solvent cast nylon-6,6 substrate, which was placed on a glass microscope slide. The reaction mixture was covered with a second glass microscope slide and irradiated with a CFL lamp placed 4 cm away without air cooling. The reaction was allowed to proceed for 18 hours before the light was turned off and the substrate removed from the glovebox. The substrate was sonicated in a mixture of methanol and water, then washed with dimethylsulfoxide overnight prior to drying under ambient conditions.

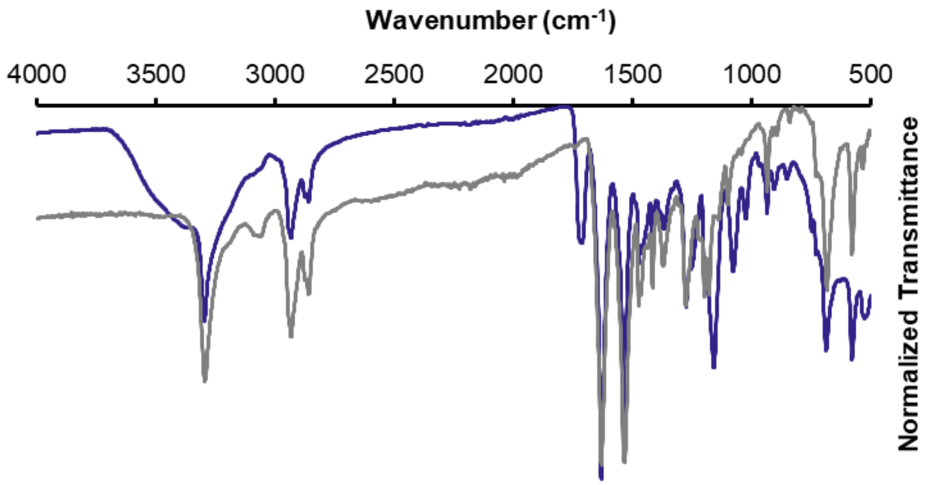

Figure S39. FT-IR spectra of nylon-6,6 before (gray) and after (purple) standard reaction conditions with HEMA.

Synthesis of nylon-6,6-g-PMA

In a nitrogen-filled glovebox, 2,2'diOMeTX (1 equiv, 0.01 mmol, 2.7 mg), BisTTC (1 equiv, 0.01 mmol, 5.5 mg), methyl acrylate (200 equiv, 2 mmol, 0.18 mL), and dioxane (0.25 mL) were combined in a one-dram vial. The solution was syringed onto a flat-solvent cast nylon-6,6 substrate, which was placed on a glass microscope slide. The reaction mixture was covered with a second glass microscope slide and irradiated with a CFL lamp placed 4 cm away without air cooling. The reaction was allowed to proceed for 18 hours before the light was turned off and the substrate removed from the glovebox. The substrate was sonicated in dichloromethane for 20 min prior to drying under ambient conditions.

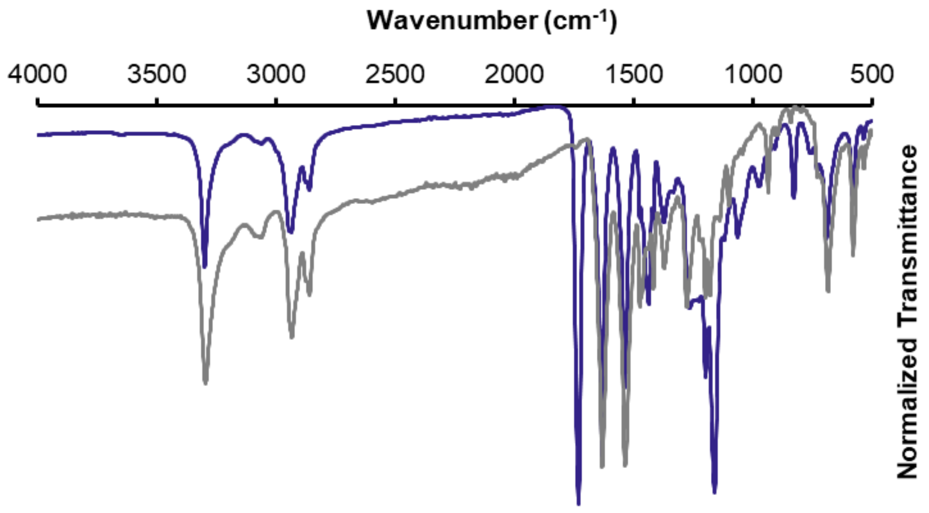

Figure S40. FT-IR spectra of nylon-6,6 before (gray) and after (purple) standard reaction conditions with MA.

Synthesis of nylon-6,6-g-PMMA

In a nitrogen-filled glovebox, 2,2'diOMeTX (1 equiv, 0.01 mmol, 2.7 mg), BisTTC (1 equiv, 0.01 mmol, 5.5 mg), methyl methacrylate (200 equiv, 2 mmol, 0.21 mL), and dioxane (0.25 mL) were combined in a one-dram vial. The solution was syringed onto a flat-solvent cast nylon-6,6 substrate, which was placed on a glass microscope slide. The reaction mixture was covered with a second glass microscope slide and irradiated with a CFL lamp placed 4 cm away without air cooling. The reaction was allowed to proceed for 18 hours before the light was turned off and the substrate removed from the glovebox. The substrate was sonicated in dichloromethane for 20 min prior to drying under ambient conditions.

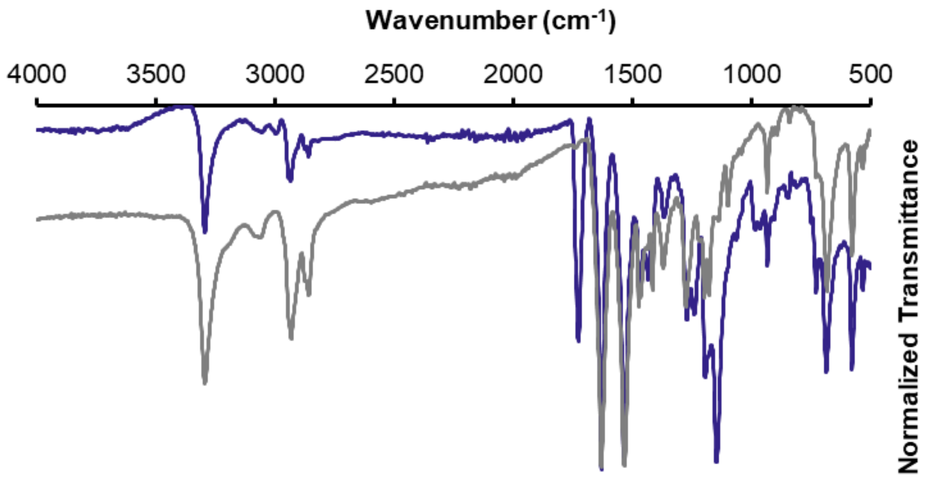

Figure S41. FT-IR spectra of nylon-6,6 before (gray) and after (purple) standard reaction conditions with MMA.

Synthesis of nylon-6,6-g-PNaA

In a nitrogen-filled glovebox, 2,2'diOMeTX (1 equiv, 0.01 mmol, 2.7 mg), BisTTC (1 equiv, 0.01 mmol, 5.5 mg), *tert*-butyl acrylate (200 equiv, 2 mmol, 0.29 mL), and dioxane (0.25 mL) were combined in a one-dram vial. The solution was syringed onto a flat-solvent cast nylon-6,6 substrate, which was placed on a glass microscope slide. The reaction mixture was covered with a second glass microscope slide and irradiated with a CFL lamp placed 4 cm away without air cooling. The reaction was allowed to proceed for 18 hours before the light was turned off and the substrate removed from the glovebox. The substrate was sonicated in dichloromethane for 20 min prior to drying under ambient conditions. The resulting nylon-6,6-*g*-P^{t}BA sample was submerged in a 0.5 M NaOH solution in 8:1 DCM:MeOH overnight and rinsed with water prior to drying under ambient conditions.

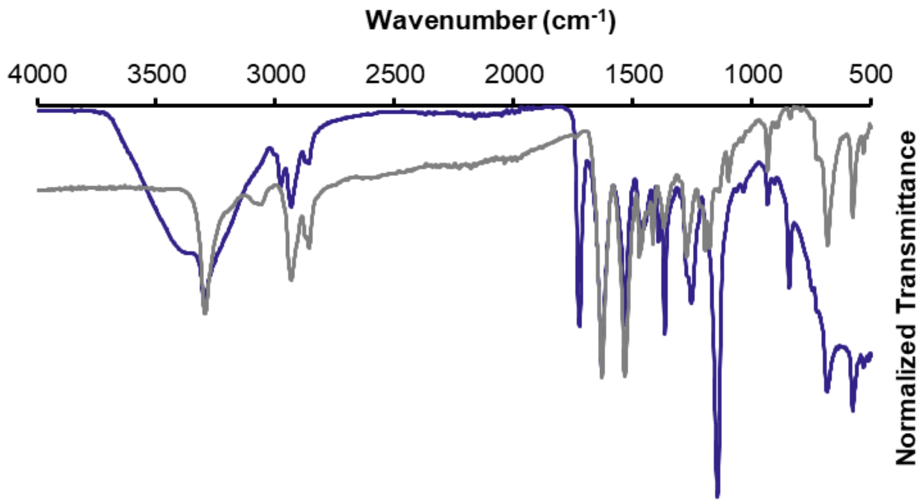

Figure S42. FT-IR spectra of nylon-6,6 before (gray) and after (purple) grafting with PNaA.

Synthesis of nylon-6,6-g-PAAm

In a nitrogen-filled glovebox, 2,2'diOMeTX (1 equiv, 0.01 mmol, 2.7 mg), BisTTC (1 equiv, 0.01 mmol, 5.5 mg), and acrylamide (200 equiv, 2 mmol, 142.2 mg) were dissolved in DMSO/dioxane (0.25 mL, 50/50 v/v) in a one-dram vial. The solution was syringed onto a flat-solvent cast nylon-6,6 substrate, which was placed on a glass microscope slide. The reaction mixture was covered with a second glass microscope slide and irradiated with a CFL lamp placed 2 cm away without air cooling. The reaction was allowed to proceed for 18 hours before the light was turned off and the substrate removed from the glovebox. The substrate was sonicated in water for 60 min and submerged in a mixture of MeOH/water (50/50 v/v) for 24 hours prior to drying under ambient conditions.

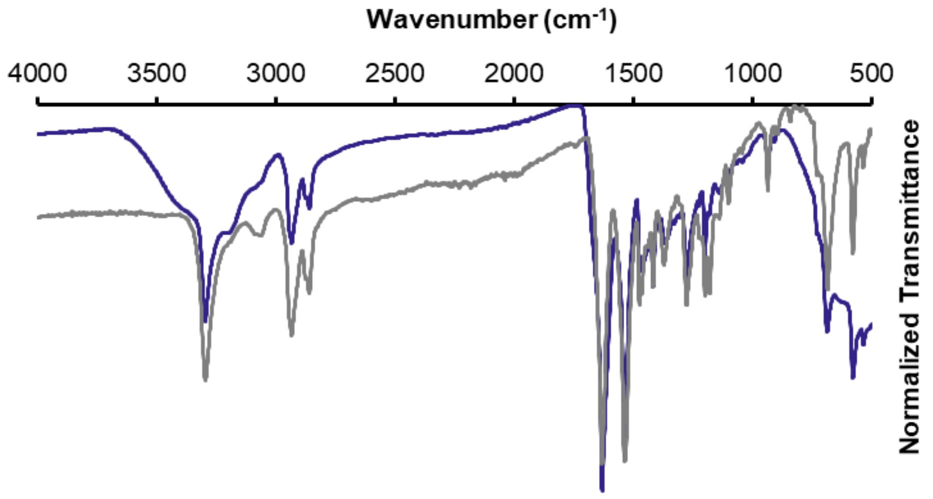

Figure S43. FT-IR spectra of nylon-6,6 before (gray) and after (purple) grafting with PAAm. FT-IR spectrum of nylon-6,6-g-PAAm was consistent with literature precedent.[5]

Synthesis of nylon-6,6-g-PNIPAm

In a nitrogen-filled glovebox, 2,2'diOMeTX (1 equiv, 0.01 mmol, 2.7 mg), BisTTC (1 equiv, 0.01 mmol, 5.5 mg), and N-isopropyl acrylamide (200 equiv, 2 mmol, 226.3 mg) were dissolved in dioxane (0.25 mL) in a one-dram vial. The solution was syringed onto a flat-solvent cast nylon-6,6 substrate, which was placed on a glass microscope slide. The reaction mixture was covered with a second glass microscope slide and irradiated with a CFL lamp placed 2 cm away without air cooling. The reaction was allowed to proceed for 18 hours before the light was turned off and the substrate removed from the glovebox. The substrate was sonicated in DCM for 40 min prior to drying under ambient conditions.

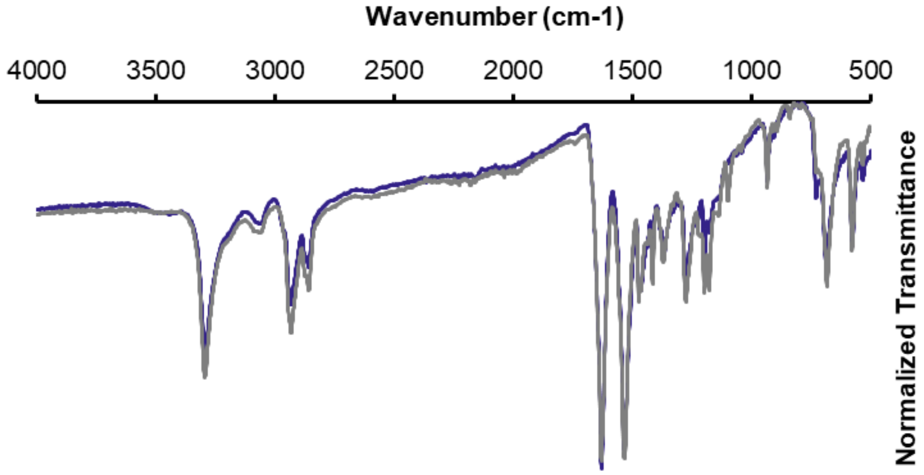

Figure S44. FT-IR spectra of nylon-6,6 before (gray) and after (purple) grafting with PNIPAm. FT-IR spectrum of nylon-6,6-g-PNIPAm was consistent with literature precedent.[6]

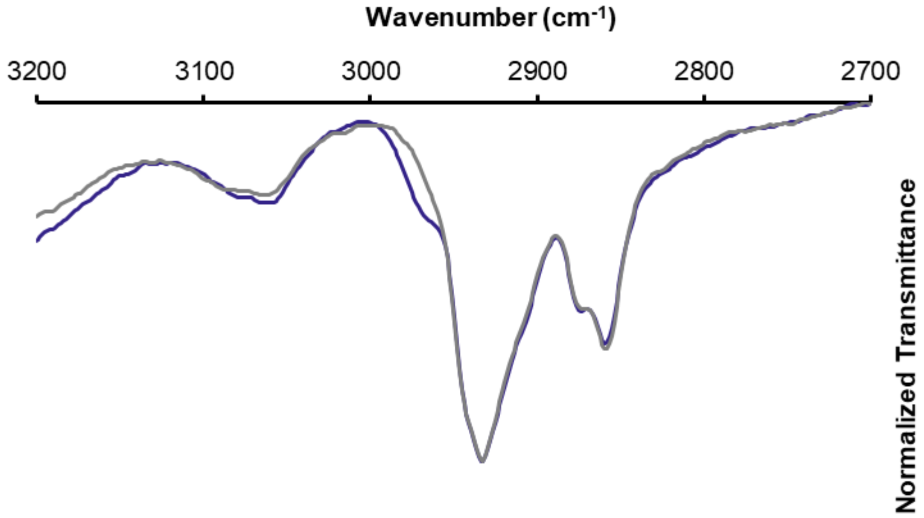

Figure S45. FT-IR spectra of nylon-6,6 before (gray) and after (purple) grafting with PNIPAm from 3200 to 2700 cm^{-1} reveal the appearance of a peak at 2970 cm^{-1} corresponding to the C–H stretch of PNIPAm.

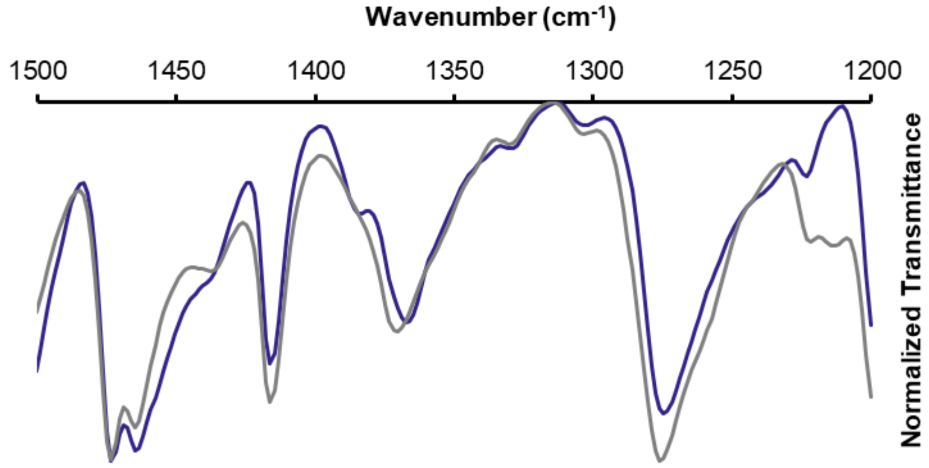

Figure S46. FT-IR spectra of nylon-6,6 before (gray) and after (purple) grafting with PNIPAm from 1500 to 1200 cm^{-1} reveal the appearance of a peak at 1385 cm^{-1} corresponding to the symmetrical C–H bending of PNIPAm.

Synthesis of nylon-6-g-P^{t}BA

In a nitrogen-filled glovebox, 2,2'diOMeTX (1 equiv, 0.01 mmol, 2.7 mg), BisTTC (1 equiv, 0.01 mmol, 5.5 mg), *tert*-butyl acrylate (200 equiv, 2 mmol, 0.29 mL), and dioxane (0.25 mL) were combined in a one-dram vial. The solution was syringed onto a flat-solvent cast nylon-6 substrate, which was placed on a glass microscope slide. The reaction mixture was covered with a second glass microscope slide and irradiated with a CFL lamp placed 4 cm away without air cooling. The reaction was allowed to proceed for 18 hours before the light was turned off and the substrate removed from the glovebox. The substrate was sonicated in dichloromethane for 20 min prior to drying under ambient conditions.

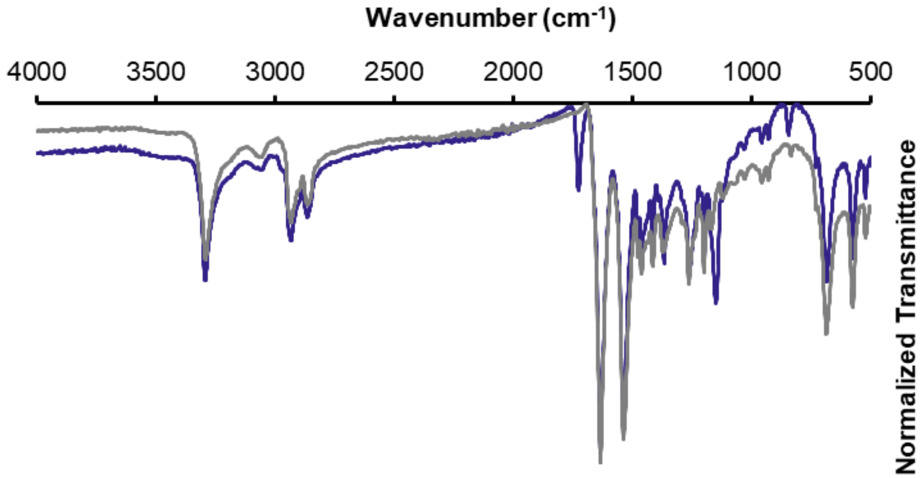

Figure S47. FT-IR spectra of nylon-6 before (gray) and after (purple) standard reaction conditions with *tert*-butyl acrylate.

Synthesis of nylon-11-g-P^{t}BA

In a nitrogen-filled glovebox, 2,2'diOMeTX (1 equiv, 0.01 mmol, 2.7 mg), BisTTC (1 equiv, 0.01 mmol, 5.5 mg), and *tert*-butyl acrylate (^{t}BA) (200 equiv, 2 mmol, 0.29 mL), and dioxane (0.25 mL) were combined in a one-dram vial. The solution was syringed onto a flat nylon-11 substrate, which was laid on a glass microscope slide. The reaction mixture was covered with a second glass microscope slide and irradiated with a CFL lamp placed 4 cm away without air cooling. The reaction was allowed to proceed for 18 hours before the light was turned off and the substrate removed from the glovebox. The substrate was sonicated in dichloromethane for 20 min prior to drying with air.

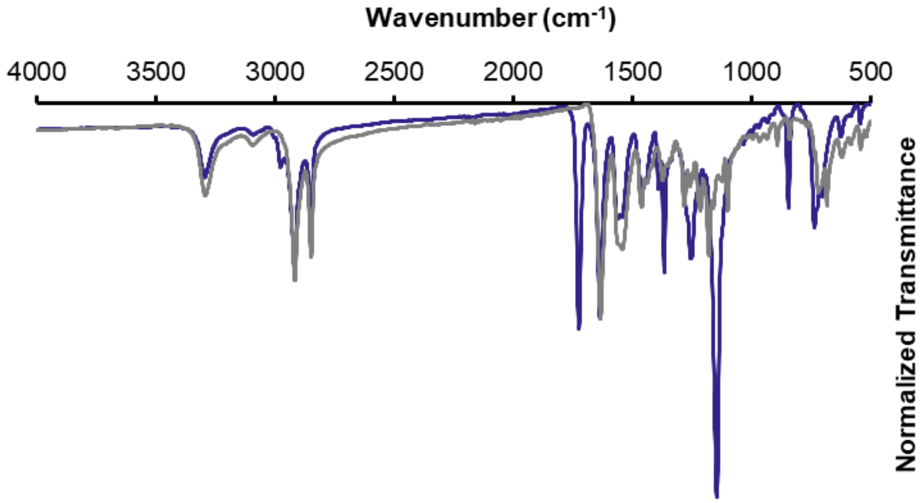

Figure S48. FT-IR spectra of nylon-11 before (gray) and after (purple) standard reaction conditions with tert-butyl acrylate.

Synthesis of Brush Polymers from nylon-6,6 fibers

In a nitrogen-filled glovebox, 2,2'diOMeTX (1 equiv, 0.01 mmol, 2.7 mg), BisTTC (1 equiv, 0.01 mmol, 5.5 mg), and *tert*-butyl acrylate (^{t}BA) (200 equiv, 2 mmol, 0.29 mL), and dioxane (0.25 mL) were combined in a one-dram vial. The solution was syringed onto a 2 cm by 2 cm sample of electrospun nylon-6,6 fibers, which was laid on a glass microscope slide. The reaction mixture was covered with a second glass microscope slide and irradiated with a CFL lamp placed 4 cm away without air cooling. The reaction was allowed to proceed for 18 hours before the light was turned off and the substrate removed from the glovebox. The substrate was sonicated in dichloromethane for 20 min prior to drying with air.

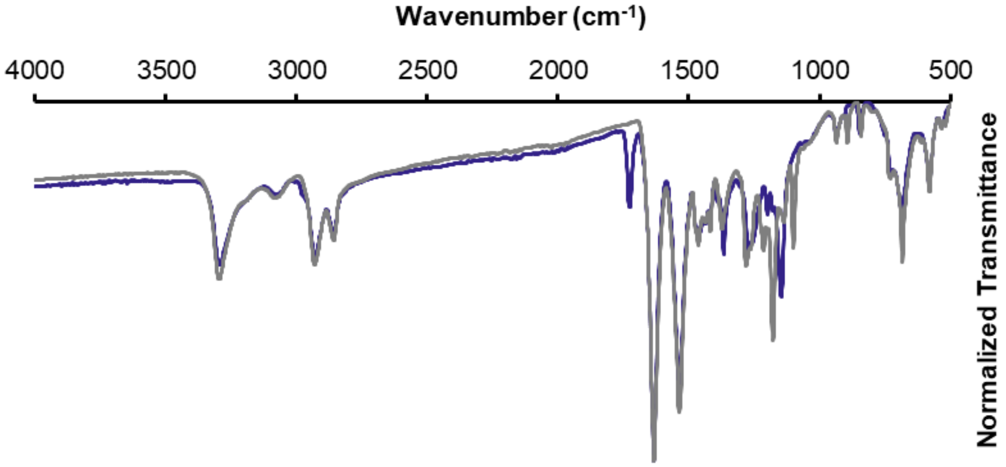

Figure S49. FT-IR spectra of electrospun nylon-6,6 before (gray) and after (purple) standard reaction conditions with *tert*-butyl acrylate.

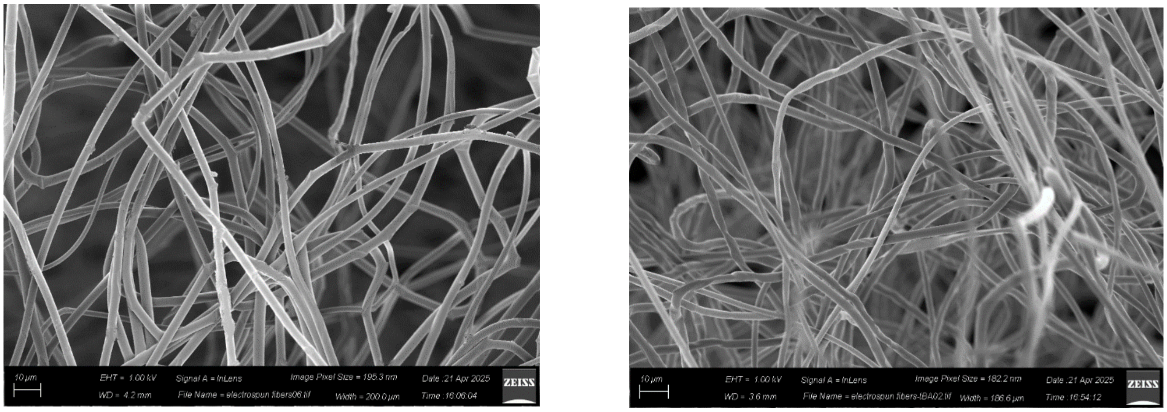

Figure S50. SEM images of electrospun nylon-6,6 fibers before (left) and after (right) standard reaction conditions with *tert*-butyl acrylate.

Synthesis of Brush Polymers from Nylon Monofilament

In a nitrogen-filled glovebox, 2,2'diOMeTX (1 equiv, 0.01 mmol, 2.7 mg), BisTTC (1 equiv, 0.01 mmol, 5.5 mg), and *tert*-butyl acrylate (^{t}BA) (200 equiv, 2 mmol, 0.29 mL), and dioxane (0.25 mL) were combined in a one-dram vial. The solution was syringed onto a 3-inch-long piece of Nylon monofilament, which was laid on a glass microscope slide. The reaction mixture was covered with a second glass microscope slide and irradiated with a CFL lamp placed 2 cm away without air cooling. The reaction was allowed to proceed for 18 hours before the light was turned off and the substrate removed from the glovebox. The substrate was sonicated in dichloromethane for 20 min prior to drying with air.

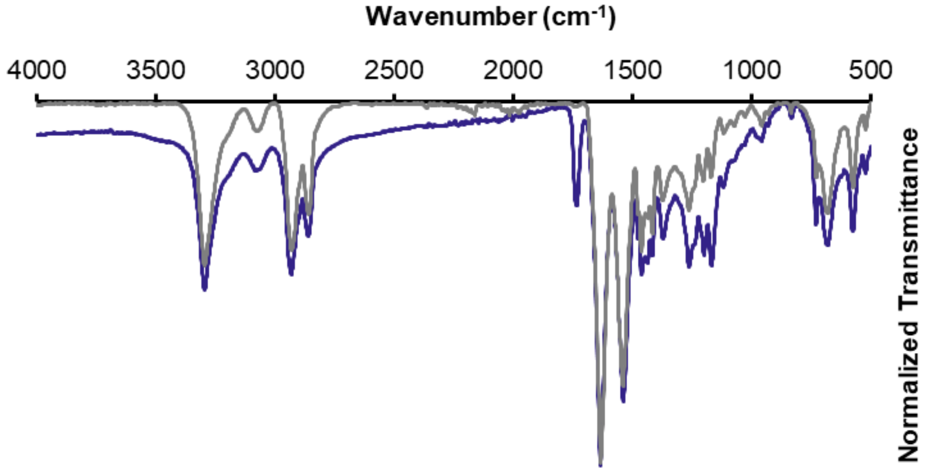

Figure S51. FT-IR spectra of nylon monofilament before (gray) and after (purple) standard reaction conditions with *tert*-butyl acrylate.

S13. Water Contact Angle Measurements

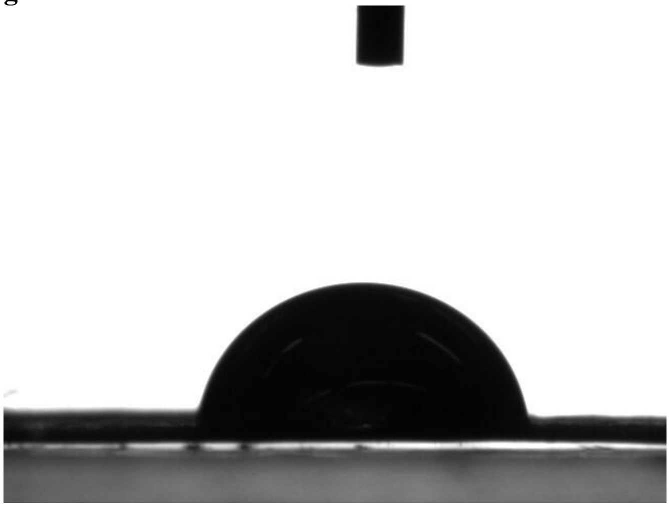

Figure S52. Representative image of a water droplet on a solvent-cast nylon-6,6 surface. The water contact angle was measured at 79 ± 2°.

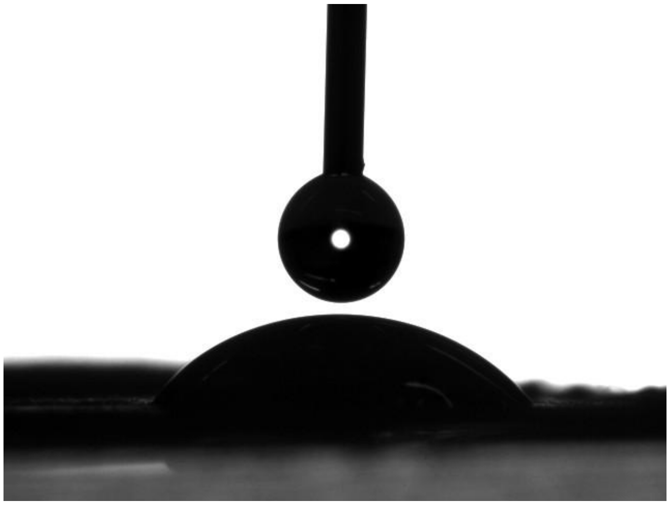

Figure S 53. Representative image of a water droplet on nylon-6.6-*g*-PHEMA. The water contact angle was measured at 43 ± 5°.

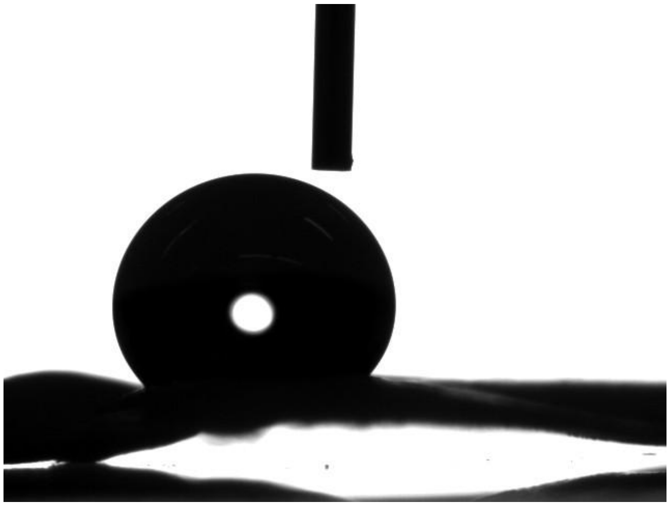

Figure S54. Representative image of a water droplet on nylon-6.6-*g*-PTMSPA. The water contact angle was measured at 131 ± 2°.

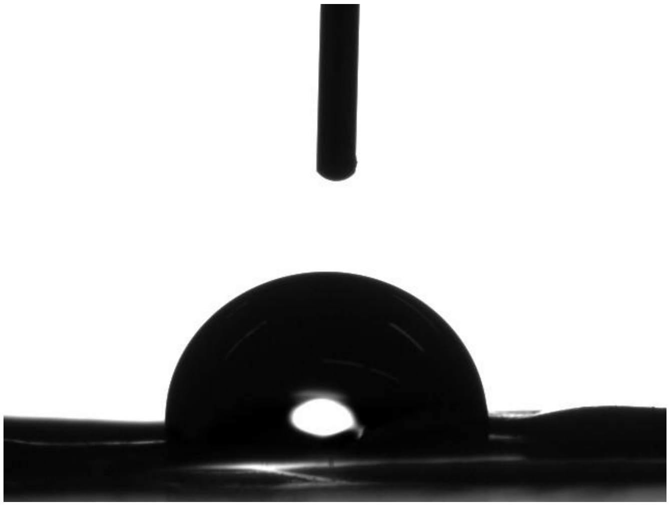

Figure S55. Representative image of a water droplet on nylon-6.6-*g*-P*ᵗ*BA. The water contact angle was measured at 88 ± 6°.

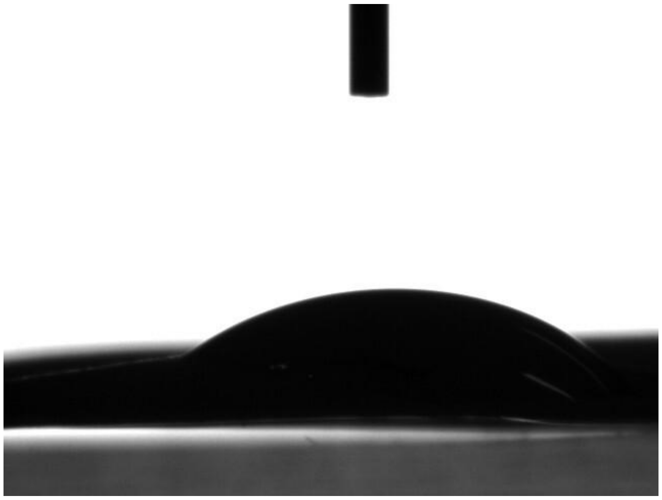

Figure S56. Representative image of a water droplet on a nylon-6.6-*g*-PEGA. The water contact angle was measured at 88 ± 6°.

S14. Patterning Experiments

Procedure for patterning P^tBA brushes from nylon-11 via SI HAT-RAFT

In a nitrogen-filled glovebox, 2,2'diOMeTX (1 equiv, 0.02 mmol, 5.5 mg), BisTTC (1 equiv, 0.02 mmol, 11 mg), methyl acrylate (100 equiv, 2 mmol, 0.36 mL) and THF (0.5 mL) were combined in a one-dram vial. The solution was syringed onto a flat-solvent cast nylon-11 substrate, which was placed on a large glass slide. The reaction mixture was covered with a fused-silica photomask patterned with 500x500 µm squares, clamped to ensure proper contact between the sample and the photomask, and irradiated with a CFL placed 4 cm away. The reaction was allowed to proceed for 3 hours before the light was turned off and the substrate removed from the glovebox. The substrate was sonicated in dichloromethane for 20 min prior to drying under ambient conditions. Patterns were visualized by irradiating with a 390 nm UV lamp and fluorescence images obtained with a 370 nm excitation wavelength.

Probing brush growth in irradiated sections of the sample

FT-IR spectra were taken to probe the degree of grafting during patterning and to ensure that grafting was not occurring without irradiation. One half of a nylon-11 sample was covered with a fused-silica photomask, and the other half was irradiated under the SI HAT-RAFT reaction conditions described above for 3 hours. A carbonyl stretch corresponding to P^tBA at 1730 cm^{-1} was apparent in the irradiated section of the sample and was not observed in the non-irradiated portions.

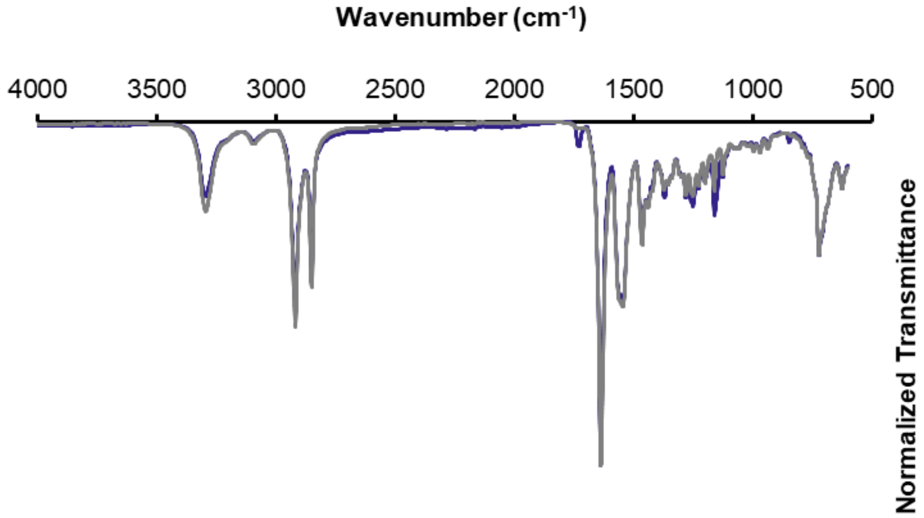

Figure S57. FT-IR spectra of irradiated (dark purple) and non-irradiated (gray) pieces of a nylon-11 film patterned with poly(*tert*-butyl acrylate) brushes.

S15. Protein Adsorption Experiments

General procedure for BSA adsorption experiments

According to a modified literature procedure,[7] pristine and grafted nylon films were soaked in PBS for 24 h at room temperature and then immersed in a PBS solution of BSA (1 mg/mL) for 3 h. Each piece was washed three times with PBS. To elute the adsorbed BSA, samples were soaked in a solution of 1 wt % sodium dodecyl sulfonate (SDS) and oscillated at 37 °C for 2 h. Following the incubation period, the samples were placed in 2 kDa MWCO dialysis tubing in distilled water for 24 h to remove excess SDS. The BSA concentration of the elution was measured with BCA and the microBCA protein assay kit, and the amount of adsorbed BSA on each was calculated. Each final result was an average of five determinations.

	R_A	BSA adsorption (µg/mL)
Nylon-6,6	-	46.0 ± 0.8
Nylon-6,6-g-PTMSPA	0.15	32.3 ± 5.2
Nylon-6,6-g-PHEMA (18h reaction time)	0.16	54.0 ± 7.7
Nylon-6,6-g-PHEMA (3h reaction time)	0.07	8.2 ± 1.5
Nylon-6,6-g-PEGA	0.08	3.9 ± 1.6

Procedure for grafting with TMSPMA for BSA adsorption experiments

In a nitrogen-filled glovebox, 2,2'diOMeTX (1 equiv, 0.01 mmol, 2.7 mg), BisTTC (1 equiv, 0.01 mmol, 5.5 mg), 3-(trimethoxysilyl)propylmethacrylate (200 equiv, 2 mmol, 0.48 mL), and dioxane (0.25 mL) were combined in a one-

dram vial. The solution was syringed onto a flat, solvent-cast nylon-6,6 substrate, which was placed on a glass microscope slide. The reaction mixture was covered with a second glass microscope slide and irradiated with a CFL lamp placed 4 cm away without air cooling. The reaction was allowed to proceed for 18 hours before the light was turned off and the substrate removed from the glovebox. The substrate was sonicated in dichloromethane for 20 min prior to drying under ambient conditions.

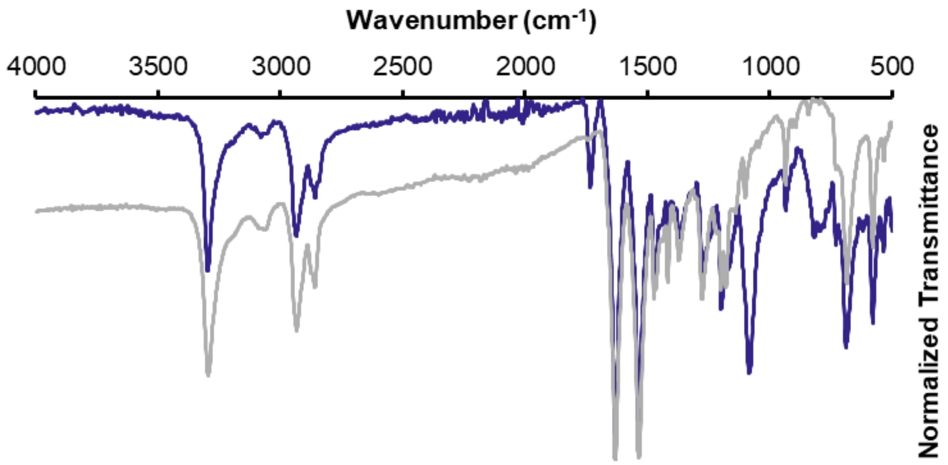

Figure S58. FT-IR of spectra of nylon-6,6 before (gray) and after (purple) reaction conditions with TMSPA.

Procedure for grafting with HEMA for BSA adsorption experiments

In a nitrogen-filled glovebox, 2,2'diOMeTX (1 equiv, 0.01 mmol, 2.7 mg), BisTTC (1 equiv, 0.01 mmol, 2.7 mg), hydroxyethyl methacrylate (200 equiv, 2 mmol, 0.24 mL), and dioxane (0.25 mL) were combined in a one-dram vial. The solution was syringed onto a flat-solvent cast nylon-6,6 substrate, which was placed on a glass microscope slide. The reaction mixture was covered with a second glass microscope slide and irradiated with a CFL lamp placed 4 cm away without air cooling. The reaction was allowed to proceed for 3 hours before the light was turned off and the substrate removed from the glovebox. The substrate was sonicated in a mixture of methanol and water, then washed with dimethylsulfoxide overnight prior to drying under ambient conditions.

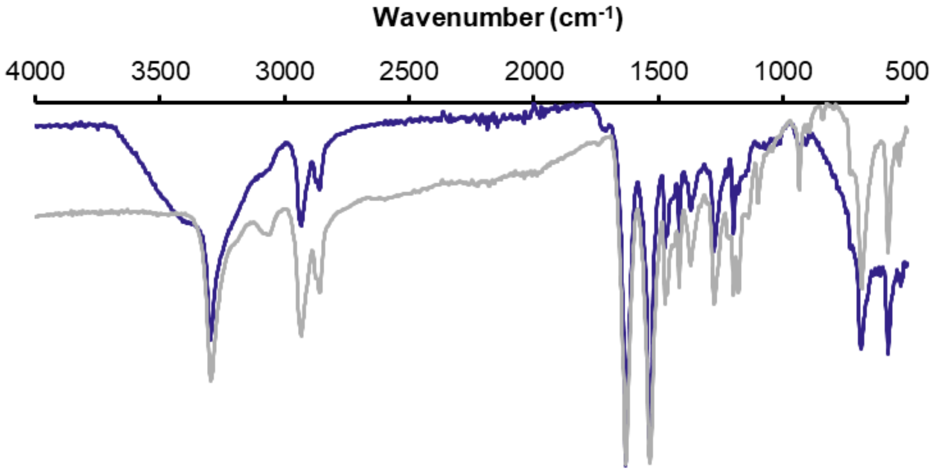

Figure S59. FT-IR of spectra of nylon-6,6 before (gray) and after (purple) 3h reaction conditions with HEMA.

Note: Samples subjected to standard reaction conditions with HEMA over 18 h did not inhibit BSA adhesion. We believe that the resulting brushes were too thick or note dense enough to inhibit protein adhesion, as has been reported previously.[8] Another possibility is that hydrogen atoms can be abstracted from PHEMA itself over long reaction times to result in a branched brush architecture, which may affect the ability to interfere with protein adhesion.[9]

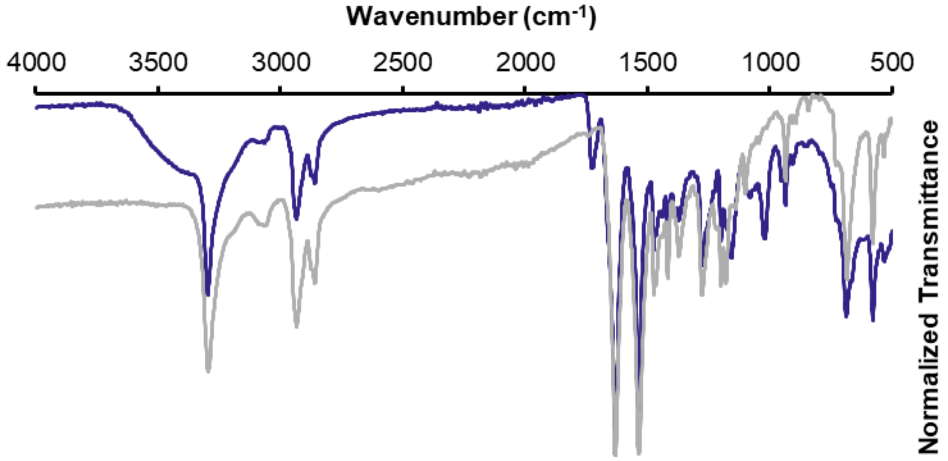

Figure S60. FT-IR of spectra of nylon-6,6 before (gray) and after (purple) standard reaction conditions with HEMA overnight.

Procedure for grafting with PEGA for BSA adsorption experiments

2,2'diOMeTX (1 equiv, 0.01 mmol, 2.7 mg), BisTTC (1 equiv, 0.01 mmol, 5.5 mg), PEG-acrylate (Mn=480) (100 equiv, 1 mmol, 0.44 mL), and dioxane (0.25 mL) were combined in a one-dram vial and subjected to three freeze-pump-thaw cycles before backfilling with nitrogen. The solution was syringed onto a flat-solvent cast nylon-6,6 substrate, which was placed on a glass microscope slide. The reaction mixture was covered with a second glass microscope slide and irradiated with a CFL lamp placed 4 cm away. The reaction was allowed to proceed for 18 hours before the light was turned off. The substrate was sonicated in water for 20 min, then acetone overnight prior to drying under ambient conditions.

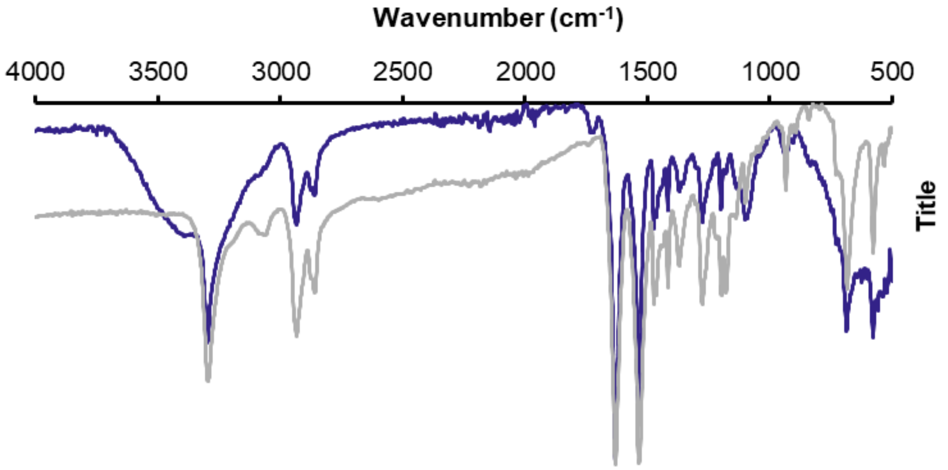

Figure S61. FT-IR of spectra of nylon-6,6 before (gray) and after (purple) reaction conditions with PEGA.

References

1 T.-T. Shih, I.-H. Hsu, J.-F. Wu, C.-H. Lin and Y.-C. Sun, *J. Chromatogr. A*, 2013, **1304**, 101–108.
2 K. Hakobyan, C. S. P. McErlean and M. Müllner, *Macromolecules*, 2020, **53**, 10357–10365.
3 E. E. Stache, V. Kottisch and B. P. Fors, *J. Am. Chem. Soc.*, 2020, **142**, 4581–4585.
4 A.-R. Cho, D. M. Shin, H. W. Jung, J. C. Hyun, J. S. Lee, D. Cho and Y. L. Joo, *J. Appl. Polym. Sci.*, 2011, **120**, 752–758.
5 S. Yu, Z. Xia, W. Kiratitanavit, S. Kulkarni, R. Sharma, D. Lu, A. B. Morgan, J. Kumar, R. Mosurkal and R. Nagarajan, *ACS Appl. Polym. Mater.*, 2024, **6**, 8876–8884.
6 T. Kajornprai, P. Katesripongsa, S. Y. Nam, Z. A. A. Hamid, Y. Ruksakulpiwat, N. Suppakarn and T. Trongsatitkul, *Polymers*, 2023, **15**, 497.
7 C. Dong, H. Wang, Z. Zhang, T. Zhang and B. Liu, *J. Colloid Interface Sci.*, 2014, **432**, 135–143.
8 C. Zhao, L. Li, Q. Wang, Q. Yu and J. Zheng, *Langmuir*, 2011, **27**, 4906–4913.
9 G. Gunkel, M. Weinhart, T. Becherer, R. Haag and W. T. S. Huck, *Biomacromolecules*, 2011, **12**, 4169–4172.
10 S. Angioni, D. Ravelli, D. Emma, D. Dondi, M. Fagnoni and A. Albini, *Adv. Synth. Catal.*, 2008, **350**, 2209–2214.
11 Y. Shen, Y. Gu and R. Martin, *J. Am. Chem. Soc.*, 2018, **140**, 12200–12209.
12 B. P. Roberts, *Chem. Soc. Rev.*, 1999, **28**, 25–35.
13 M. Pérez-Perrino, R. Navarro, O. Prucker and J. Rühe, *Macromolecules*, 2014, **47**, 2695–2702.
14 O. Prucker, C. A. Naumann, J. Rühe, W. Knoll and C. W. Frank, *J. Am. Chem. Soc.*, 1999, **121**, 8766–8770.
15 Y. Nakamura, R. Lee, M. L. Coote and S. Yamago, *Macromol. Rapid Commun.*, 2016, **37**, 506–513.
